# Metabolic profiling of antigen-specific CD8^+^ T cells by spectral flow cytometry

**DOI:** 10.1016/j.crmeth.2025.101185

**Published:** 2025-09-26

**Authors:** Nils Mülling, J. Fréderique de Graaf, Graham A. Heieis, Kristina Boss, Benjamin Wilde, Bart Everts, Ramon Arens

**Affiliations:** 1 Department of Immunology, Leiden University Medical Center, 2333 ZA Leiden, the Netherlands; 2 Department of Nephrology, University Hospital Essen, University Duisburg-Essen, 45147 Essen, Germany; 3 Leiden University Center of Infectious Diseases, Leiden University Medical Center, 2333 ZA Leiden, the Netherlands

**Keywords:** T cell, metabolism, antigen-specific, spectral flow cytometry

## Abstract

Cytotoxic CD8^+^ T cells are essential mediators of immune responses against viral infections and tumors. Upon antigen encounter, antigen-specific CD8^+^ T cells undergo clonal expansion and produce effector cytokines, processes that require dynamic metabolic adaptation. However, profiling antigen-specific T cells at single-cell resolution remains technically challenging. We present a spectral flow cytometry-based workflow enabling metabolic profiling of antigen-specific CD8^+^ T cells identified via major histocompatibility complex (MHC) class I tetramers or CD137 upregulation. The approach integrates the analysis of metabolic protein expression to infer pathway activity, uptake of fluorescent probes to measure functional metabolism and metabolite utilization, and assays evaluating cellular energy metabolism. Applied to human and mouse samples, this method defined the metabolic profiles of cytomegalovirus-, SARS-CoV-2-, and tumor-specific CD8^+^ T cells across distinct activation states and tissues. By detailing each component of the workflow, we provide practical guidance for applying metabolic spectral flow cytometry to dissect disease mechanisms and therapeutic responses.

## Highlights

• Spectral flow cytometry enables single-cell metabolic profiling of antigen-specific T cells
• Enzyme and transporter expression reveal the activity of key metabolic pathways
• SCENITH and fluorescent metabolic probes uncover functional metabolic states
• High-dimensional analysis combines metabolic and phenotypic profiling of CD8^+^ T cells

## Motivation

Understanding T cell metabolism is key to unraveling immune responses in chronic viral infections and cancer and to improving immunotherapies. However, conventional metabolic assays lack the sensitivity to analyze rare, antigen-specific T cells at single-cell resolution. To overcome this, we developed a spectral flow cytometry-based workflow for the comprehensive metabolic profiling of antigen-specific CD8 ^+^ T cells. By integrating metabolic protein staining, fluorescent probes, and functional energy state indicators with MHC class I tetramers or CD137 upregulation, our method enables *ex vivo* analysis of human and mouse CD8^+^ T cells across blood and tissues.

## Graphical abstract

**Figure:**
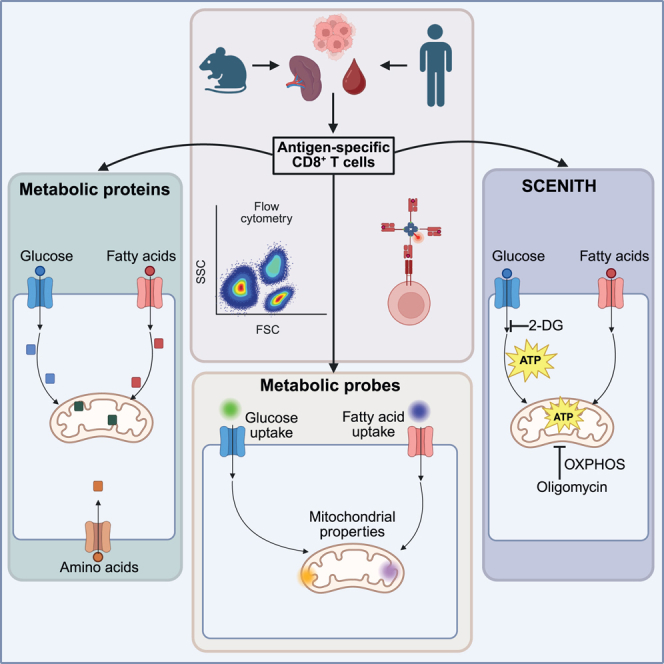

## Teaser

Mülling et al. present a spectral flow cytometry-based workflow to profile the metabolism of rare, antigen-specific CD8^+^ T cells. This approach enables insights into how these immune cells adapt their energy use during infection and cancer, offering tools to better understand disease and guide therapies.

## Introduction

CD8^+^ T cells are critical components of the adaptive immune system, playing a central role in in the defense against viral infections and cancer.^1^ CD8^+^ T cells recognize pathogen- or tumor-derived peptides presented by major histocompatibility complex (MHC) class I molecules via their T cell receptor (TCR). Upon antigen encounter, naive CD8^+^ T cells become activated and undergo clonal expansion, followed by contraction and memory formation.^2^ Each of these phases is characterized by distinct transcriptional, epigenetic, and metabolic reprogramming.^3^

Naive CD8^+^ T cells are metabolically quiescent and primarily depend on oxidative phosphorylation (OXPHOS) for energy. In contrast, activated effector T cells have high demands of energy production to proliferate and execute their effector functions and shift to aerobic glycolysis, a less efficient but faster ATP-generating pathway.^4^ In addition to energy production, proliferating cells require increased biosynthesis of proteins, fatty acids, and cholesterol. During the transition to memory, CD8^+^ T cells progressively revert to enhanced reliance on OXPHOS, partly driven by increased fatty acid oxidation (FAO).^4^

In contrast to acute infections, chronic infections and cancer lead to ongoing TCR-mediated activation, resulting in dysfunctional antigen-specific CD8^+^ T cells. These exhausted CD8^+^ T cells exhibit impaired effector function, reduced proliferative capacity, and distinct metabolic alterations.^5^^,^^6^^,^^7^^,^^8^^,^^9^ While progenitor exhausted CD8^+^ T cells are marked by decreased glycolytic capacity with compensatory upregulated FAO, terminally exhausted CD8^+^ T cells display mitochondrial dysfunction and rely predominantly on glycolysis.^5^^,^^10^ A detailed understanding of the metabolic states and (dys-)function of antigen-specific CD8^+^ T cells is therefore critical for developing targeted therapies aimed at restoring or enhancing T cell functionality in chronic infections and cancer.^11^

Over the past decades, several techniques have emerged to study immunometabolism. Approaches such as metabolomics and extracellular flux analysis provide powerful tools to study metabolic alterations of immune cell populations, but they require high cell numbers as input and lack single-cell resolution. As a result, studying rare immune subsets remains challenging without prior cell manipulation such as *in vitro* expansion, which can alter the metabolic properties substantially.^12^ To address these limitations, cytometry-based techniques like Met-flow^13^^,^^14^ and single-cell energetic metabolism by profiling translation inhibition (SCENITH)^15^ were developed to overcome the prescribed hurdles to study rare cell populations, as such methods enable metabolic analysis at single-cell resolution. Building on these advances, we present a spectral flow cytometry-based workflow that enables the metabolic profiling of antigen-specific CD8^+^ T cells at single-cell resolution without the need for prior cell purification. Our approach integrates the measurement of metabolic enzyme and transporter expression, metabolic probes to study glucose and fatty acid uptake and mitochondrial properties, and the SCENITH assay, which allows to study energy metabolism by profiling translation inhibition. Furthermore, these methods are compatible with concurrent staining of phenotypic markers, providing additional insight into the metabolic-phenotypic landscape of antigen-specific T cells. We applied these methods to human and murine samples derived from blood, spleen, and tumor tissue to characterize cytomegalovirus-, SARS-CoV-2-, and tumor-specific CD8^+^ T cells.

## Results

### Spectral flow cytometry-based metabolic profiling of antigen-specific CD8^+^ T cells

The spectral flow cytometry-based workflow described here enables the metabolic profiling of antigen-specific CD8^+^ T cells by combining their detection using MHC class I tetramers or CD137 upregulation with single-cell resolution assays of metabolic function.

**Figure 1:**
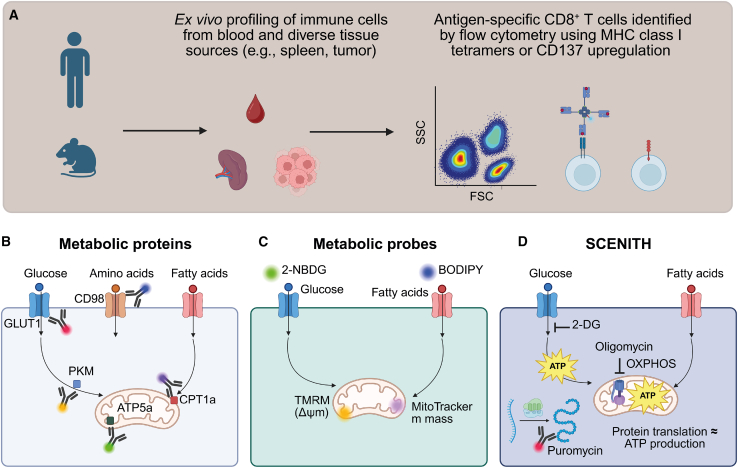
Spectral flow cytometry-based metabolic profiling of antigen-specific CD8^+^ T cells (A) Workflow overview. Single-cell suspensions were prepared from blood or tissue for spectral flow cytometry analysis. MHC class I tetramers or CD137 expression was used to identify antigen-specific CD8^+^ T cells and combined with staining for phenotypic markers, metabolic proteins, probes, and other features. (B) Staining with fluorescently labelled antibodies against metabolic proteins enables the analysis of key metabolic pathways with single-cell resolution. (C) Uptake of fluorescent metabolic probes reveals glucose and fatty acid uptake, as well as mitochondrial properties (e.g., mitochondrial mass and membrane potential). (D) SCENITH assay enables functional profiling of metabolic activity based by measuring puromycin incorporation in the presence of inhibitors of glycolysis (by 2-DG) and oxidative phosphorylation (OXPHOS; by oligomycin). Figure created with www.biorender.com.

The use of MHC class I tetramers is an established technique to study antigen-specific CD8^+^ T cells by flow or mass cytometry.^16^ Using this approach, we examined CD8^+^ T cells specific against human cytomegalovirus (HCMV), murine cytomegalovirus (MCMV), and SARS-CoV-2 derived from blood and tissue samples (Figure 1A). In addition, we profiled tumor-specific CD8^+^ T cells derived from tumor tissue. The antigen-specific CD8^+^ T cell populations were assessed for metabolic protein expression (Figure 1B), uptake of fluorescent metabolic probes (Figure 1C), and metabolic capacities and dependencies by SCENITH (Figure 1D).

### Metabolic profiling of antigen-specific CD8^+^ T cells in blood and tissues

Peripheral blood mononuclear cells (PBMCs) from HCMV-seropositive healthy donors were cryopreserved in liquid nitrogen until use. For metabolic analysis, PBMCs were thawed and rested for 2 h in a medium supplemented with DNase I to reduce cell clumping and improve single-cell suspension quality. This post-thaw resting phase is vital for T cell recovery and should be at least 1 h but can be extended to overnight without adverse effects. Fresh PBMCs can be processed for staining immediately, without the need for a resting period. The gating strategy for flow cytometric analysis is provided in Figure S1A. Notably, the duration of the resting phase (1 h vs. overnight) did not affect the staining intensity of metabolic proteins (shown for CPT1a; Figure S1B).

The flow cytometry staining panels were optimized to investigate multiple metabolic pathways, including a) amino acid uptake, identified by a component of the neutral amino acid transporter (CD98); b) glycolysis, identified by the glucose transporter GLUT1 and the rate-limiting enzyme pyruvate kinase (PKM); c) the pentose phosphate pathway, identified by glucose-6-phosphate dehydrogenase (G6PD); d) FAO, identified by the rate-limiting enzyme carnitine palmitoyl transferase 1a (CPT1a); e) the tricarboxylic acid (TCA) cycle, identified by succinate dehydrogenase A (SDHA); and f) mitochondrial respiration, identified by ATP synthase F1 subunit alpha (ATP5a). All antibodies were titrated to achieve optimal resolution, and multicolor panels were optimized using fluorescence minus one (FMO) controls for the metabolic antibodies, as exemplified in Figures S1C and S1D.

The selected metabolic targets cover key pathways involved in T cell metabolism, enabling a comprehensive overview of their metabolic state. Nonetheless, panels can be further expanded to include additional processes such as glutaminolysis (e.g., via glutaminase [GLS]), fatty acid synthesis (e.g., via fatty acid synthase [FASN]), or cholesterol biosynthesis (e.g., via farnesyl-diphosphate farnesyltransferase 1 [FDFT1]). Example staining for these targets is shown in Figure S1E. While the abundance of metabolic proteins does not directly equate to metabolic activity, previous studies have demonstrated a strong correlation between protein expression levels and functional metabolic readouts.^13^

**Figure 2:**
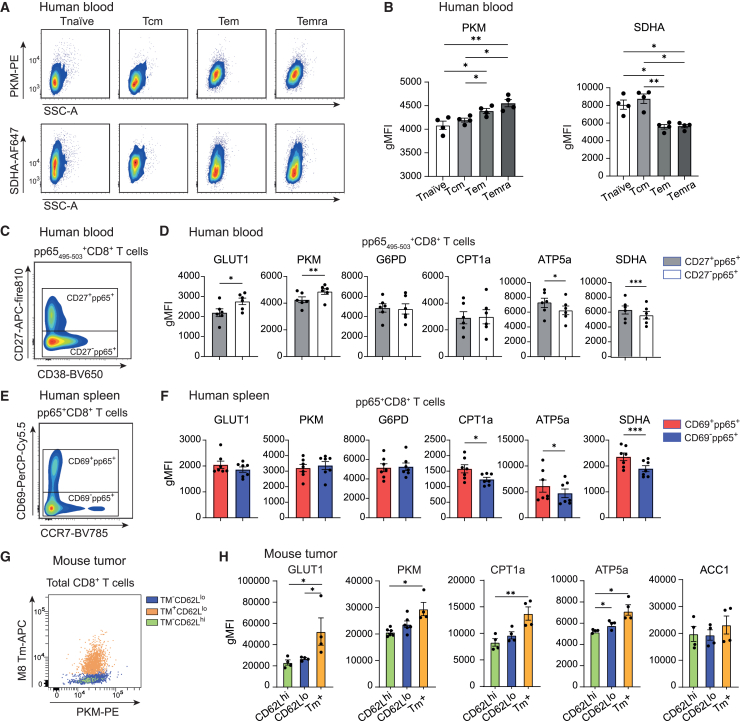
Metabolic profiling of antigen-specific CD8^+^ T cells in blood and tissues (A) Representative flow cytometry plots showing the expression of metabolic proteins in circulating CD8^+^ T cell subsets from a human donor. (B) Quantification of PKM and SDHA expression (geometric mean fluorescence intensity [gMFI]) in circulating CD8^+^ T cell populations from human donors. (C) Gating strategy to distinguish CD27^+^ and CD27^−^ pp65_495–503_-specific CD8^+^ T cells in human blood. Shown is a representative flow cytometry plot gated on pp65_495–503_-specific-CD8^+^ T cells identified by the MHC class I tetramer HLA-A∗02-HCMV pp65_495–503_. (D) Expression (gMFI) of metabolic proteins in CD27^+^ and CD27^−^ pp65_495–503_-specific CD8^+^ T cells from HCMV-positive donors (*n* = 6). (E) Gating strategy for CD69^+^ and CD69^−^ pp65_495–503_-specific CD8^+^ T cells in human spleen. Shown is a representative flow cytometry plot gated on pp65^+^CD8^+^ T cells identified by two APC-labeled MHC class I tetramers: HLA-A∗02-HCMV pp65_495–503_ (NLVPMVATV) and HLA-A∗01-HCMV pp65_363–373_ (YSEHPTFTSQY). (F) Expression (gMFI) of metabolic proteins in CD69^+^ and CD69^−^ splenic pp65-specific CD8^+^ T cells (*n* = 7 HCMV-positive spleen donors). (G) Representative flow cytometry plot showing PKM expression in CD62L^hi^, CD62L^lo^, and M8-specific CD8^+^ T cells from MC-38 tumor-bearing mice. Tumor-specific CD8^+^ T cells were identified with the H-2K_b_ tetramer M8_604–611_. (H) Expression (gMFI) of GLUT1, PKM, CPT1a, ATP5a, and ACC1 in CD62L^hi^, CD62L^lo^, and M8-specific CD8^+^ T cells isolated from MC-38 tumor-bearing mice. Dots represent individual mice. Data are shown as mean ± SEM. Statistical analysis was performed by repeated-measures ANOVA with Tukey’s multiple comparisons test or two-tailed paired t test. ∗*p* < 0.05; ∗∗*p* < 0.01; ∗∗∗*p* < 0.001. See also Figure S2 and Table S1.

As an example, we analyzed metabolic protein expression across distinct CD8^+^ T cell subsets: naive (T_naive_), central memory (T_cm_), effector memory (T_em_), and effector memory re-expressing CD45RA (T_emra_) cells (Figure S2A). In line with previous findings, resting T_naive_ cells are metabolically quiescent and primarily rely on OXPHOS to meet their energy demands.^4^ Consistently, the abundance of SDHA is enhanced compared to T_em_ and T_emra_ cells. In contrast, T_em_ and T_emra_ subsets rely more on glycolysis, as indicated by an increased expression of PKM. T_cm_ cells differ from the effector memory subsets as they display a pattern more comparable to T_naive_ cells, with increased reliance on OXPHOS compared to glycolysis^4^^,^^17^^,^^18^ (Figures 2A and 2B).

To assess the impact of cryopreservation, we compared fresh and thawed PBMCs from the same donor. Staining intensities were comparable between conditions, and metabolic differences between subsets (e.g., T_naive_ and T_em_) remained preserved (Figure S2B). It is important to note that outcomes may vary depending on the specifics of the cryopreservation and thawing protocols used, as well as the technical expertise of the operator.

To characterize the antigen-specific CD8^+^ T cells specific for HCMV, we used HLA-A2 tetramers loaded with pp65 peptide, an immunodominant antigenic peptide^19^ (Figure S2C). In addition, phenotypical markers were included in the panel to enable the association of metabolic profiles with the differentiation status of antigen-specific CD8^+^ T cells. As an example, we demonstrate the difference in metabolic profiles between pp65-specific CD8^+^ T cells with or without expression of the costimulatory receptor and differentiation marker CD27 (Figures 2C and 2D; Table S1). HCMV-specific CD8^+^ T cells lacking CD27 expression, classifying more differentiated cells, were marked by an increased abundance of GLUT1 and PKM along with a reduced expression of ATP5a and SDHA, indicating an increased glycolytic reliance compared to CD27^+^ pp65-specific CD8^+^ T cells. No differences were observed in the abundance of G6PD and CPT1a (Figures 2D and S2D).

A key consideration when analyzing metabolic protein expression by (spectral) flow cytometry is that differences are typically reflected as shifts in fluorescence intensity, rather than as clearly distinct positive or negative populations. Consequently, it is often not feasible to define discrete gates for “positive” or “negative” expression. We therefore recommend using the geometric mean fluorescence intensity (gMFI) as a quantitative readout of metabolic protein abundance. It is also important to note that the staining intensity of metabolic protein expression can be influenced by the total number of cells present in each sample. For example, we observed a reduction in the gMFI of cytochrome c, GLUT1, PKM, and CPT1a of CMV-specific CD8^+^ T cells when the numbers of PBMCs stained were increased from 0.5 to 1 × 10^6^ cells (Figure S2E). These findings underscore the importance of using equal cell numbers across samples to ensure reliable comparisons of metabolic protein expression.

To assess whether metabolic pathway analysis based on protein expression can also be applied to lymphocytes isolated from tissues, we evaluated this method using human spleen samples obtained from deceased organ donors. Frozen spleen-derived lymphocytes were thawed and rested overnight in a culture medium containing DNase I. After resting, we analyzed the properties of tissue-resident (Trm; identified as CD69^+^)^20^ and circulating CD69^−^ pp65-specific CD8^+^ T cells (Figure 2E). The analysis of metabolic protein abundances showed no differences between circulating and tissue-resident cells in glucose metabolism-related protein expression (GLUT1, PKM, and G6PD). In contrast, the abundances of CPT1a, ATP5a, and SDHA were increased in HCMV-specific Trm cells, indicating a preference for FAO-fueled mitochondrial respiration (Figure 2F). Moreover, this approach is compatible with collagenase-treated tumor tissue, as demonstrated by the use of the H-2K_b_ tetramer M8_604–611,_ to identify tumor-specific CD8^+^ T cells isolated from MC38 tumor-bearing. In this setting, tumor-specific CD8^+^ T cells exhibited signs of enhanced metabolic activity, including increased GLUT1, PKM, CPT1a, and ATP5a, compared to control mice^21^ (Figures 2G and 2H).

### Metabolic profiling of antigen-specific CD8^+^ T cells upon activation

To compare metabolic protein expression between resting and activated HCMV-specific CD8^+^ T cells, PBMCs from human donors were either stimulated with pp65_495–503_ peptide for 6 days or left untreated, and the abundance of metabolic proteins was analyzed. To control for culture-related effects, unstimulated cells were maintained under identical conditions for 6 days. The use of total PBMCs preserved high viability in both stimulated and unstimulated cell cultures (Figure S3A). However, prolonged culture of purified populations, such as isolated CD8^+^ T cells, often results in substantial viability loss without stimulation. In such cases, freshly isolated cells may serve as more appropriate unstimulated controls. To account for culture-induced variation, we recommend including paired control samples from the same donor for both conditions. In addition, following activation, the identification of antigen-specific CD8^+^ T cells using MHC class I tetramers can be compromised due to TCR internalization. However, during this period, stimulated cells can be detected by the upregulation of the activation marker CD137^22^ (Figure S3B). As the TCR is recycled back to the cell surface over time, tetramer staining becomes feasible again. In our experimental setup, HCMV-specific CD8^+^ T cells were identified by MHC class I tetramers both in the resting phase and after 6 days of stimulation, while CD137 expression was used to detect antigen-specific cells at day 3 post-stimulation (Figure S3B).

**Figure 3:**
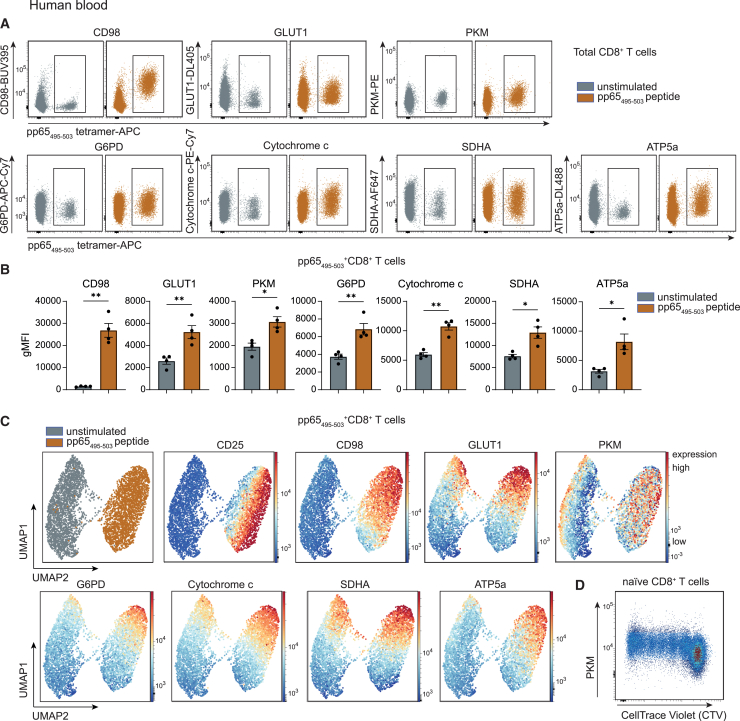
Metabolic profiling of antigen-specific CD8^+^ T cells upon activation (A) Representative flow cytometry plots gated on human CD8^+^ T cells from blood. Expression of metabolic proteins is shown vs. pp65_495–503_ tetramer staining. Gate indicates pp65_495–503_^+^ cells. Gray: unstimulated; orange: stimulated for 6 days with pp65_495–503_ peptide. (B) Expression (gMFI) of metabolic proteins in unstimulated and stimulated pp65_495–503_-specific CD8^+^ T cells from HCMV-positive donors (*n* = 4). Gray: unstimulated; orange: stimulated for 6 days with pp65_495–503_ peptide. (C) UMAP visualization of unstimulated and 6-day stimulated pp65_495–503_-specific CD8^+^ T cells. Expression of CD25 and selected metabolic proteins is overlaid. (D) Naive CD8^+^ T cells were isolated, labeled with cell trace violet (CTV) and stimulated with anti-CD3 and anti-CD28 monoclonal antibodies for 6 days. Representative flow cytometry plot displays PKM expression vs. CTV dilution. Data are shown as mean ± SEM. Statistical analysis was performed using two-tailed paired t tests. ∗*p* < 0.05; ∗∗*p* < 0.01. See also Figure S3.

In steady state without stimulation, the expression of CD98, a component of the large neutral amino acid transporter, was minimal, while that of other metabolic proteins GLUT1, PKM, G6PD, cytochrome c, SDHA, and ATP5a was detectable (Figures 3A and 3B). Following activation, we observed a marked upregulation of the nutrient transporters CD98 (extracellular) and GLUT1 (intracellular), along with increased expression of intracellular metabolic enzymes. This indicates that both the import and consumption of glucose and amino acids, as well as their downstream metabolic processing, are actively induced upon T cell activation (Figures 3A and 3B). High-dimensional visualization by UMAP enables to analyze the complexity of the protein expression data with single-cell resolution. The activation marker CD25, which is still expressed after 6 days of stimulation, clearly separates unstimulated from antigen-stimulated CD8^+^ T cells, and within the stimulated population, the metabolic proteins substantially overlap, thereby indicating synchronized metabolic processes (Figure 3C).

Metabolic protein staining is compatible with proliferation dyes, enabling the analysis of metabolic protein expression in relation to cell division (Figures 3D and S3C). When sufficient cell numbers are available, this approach can also be applied to antigen-specific CD8^+^ T cells. The combination of proliferation tracking and metabolic profiling adds an additional layer of insight, allowing researchers to assess how metabolic programs evolve during clonal expansion. For example, we observed that the expression of PKM, ATP5a, and CPT1a increased upon cell cycle progression (Figures 3D and S3C), highlighting dynamic metabolic adaptation during T cell activation and proliferation.

### Metabolic profiling detects differences between antigen-specific CD8^+^ T cell populations directed against distinct viruses in the same host

**Figure 4:**
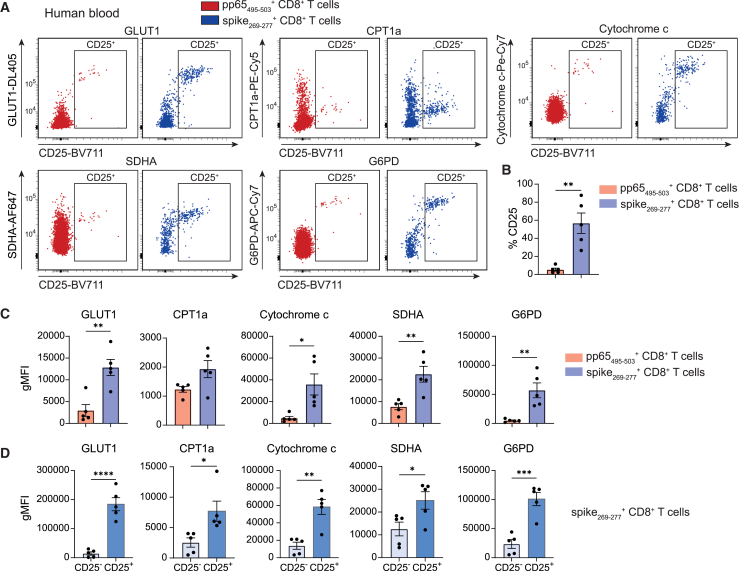
Metabolic profiling distinguishes antigen-specific CD8^+^ T cell populations targeting different viruses within the same host PBMCs were obtained from individuals with mild acute SARS-CoV-2-infection and positive HCMV serology (A) Representative flow cytometry plots showing the expression of metabolic proteins vs. CD25 in pp65_495–503_- (red) and spike_269–277_-specific (blue) CD8^+^ T cells. Gates indicate CD25^+^ cells. (B) Frequency of CD25^+^ cells among pp65_495–503_- and spike_269–277_-specific CD8^+^ T cells (*n* = 5). (C) Expression (gMFI) of metabolic proteins in pp65_495–503_- (red) and spike_269–277_-specific (blue) CD8^+^ T cells from individual donors (*n* = 5). (D) Expression (gMFI) of metabolic proteins in CD25^−^ and CD25^+^ spike_269–277_-specific CD8^+^ T cells (*n* = 5). Data are presented as mean ± SEM. Statistical analysis was performed by two-tailed paired t tests. ∗*p* < 0.05; ∗∗*p* < 0.01, ∗∗∗*p* < 0.001. See also Figure S4.

To evaluate if this approach is also capable of reflecting metabolic differences between virus-specific CD8^+^ T cell populations directed against different viruses in the same host, we analyzed PBMCs from HCMV-positive healthy individuals with mild acute SARS-CoV-2 infection. When comparing the total populations of HCMV- and SARS-CoV-2-specific CD8^+^ T cells, we observed higher expression of metabolic proteins in SARS-CoV-2-specific CD8^+^ T cells, including those related to glucose uptake (GLUT1), mitochondrial respiration (cytochrome c and SDHA), and the pentose-phosphate pathway (G6PD; Figures 4A and 4B). Notably, this was mainly due to an increased fraction of activated CD25-positive cells within the SARS-CoV-2-specific CD8^+^ T cells (Figures 4A and 4B). The fraction of CD25-positive CD8^+^ T cells in the subset of HCMV-specific CD8^+^ T cells was not present in all donors analyzed (Figure S4). Moreover, this assay is capable of distinguishing between resting memory and CD25^+^ activated effector T cells within the same antigen-specific CD8^+^ T cell population (Figure 4D). Thus, antigen-specific CD8^+^ T cells directed against distinct viruses can be simultaneously investigated in the same host for their metabolic activity. Moreover, depending on the population of interest, flow cytometry panels can be customized to include over 20 markers, combining metabolic and lineage-specific proteins. This allows for simultaneous analysis of antigen-specific CD8^+^ T cells alongside other immune cell types such as CD4^+^ T cells and B cells (Figure S5; Table S1).

### The infectious dose impacts the metabolism of antigen-specific CD8^+^ T cells *in vivo*

**Figure 5:**
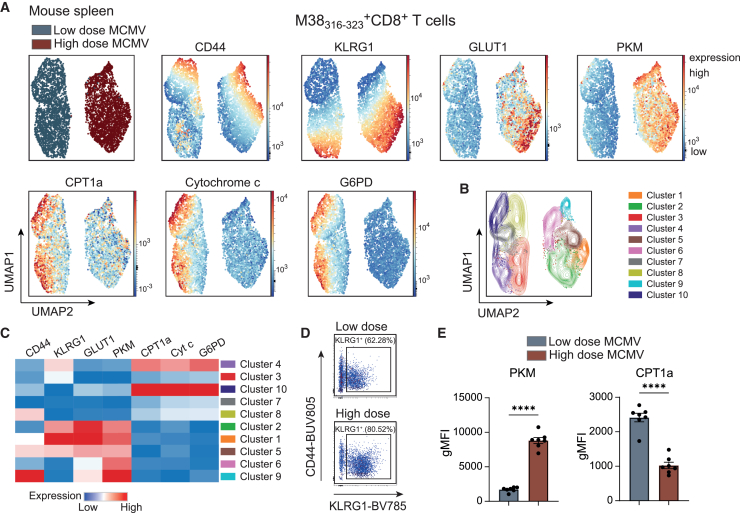
The infectious dose modulates the metabolic phenotype of antigen-specific CD8^+^ T cells *in vivo* C57BL/6 mice were infected intraperitoneally with a low (2 × 10^2^ PFU) or high (2 × 10^4^ PFU) dose of MCMV. Spleens were harvested on day 20 post-infection and analyzed by spectral flow cytometry (*n* = 7 per group). (A) UMAP analysis of down-sampled equally M38_316-323_-specific CD8^+^ T cells. Blue: low-dose infection. Red: high-dose infection. Expression of selected phenotypic and metabolic proteins is shown. (B) FlowSOM consensus meta-clustering (k = 10) based on markers displayed in (A). (C) Hierarchically clustered heatmap of phenotypic marker and metabolic protein expression of the clusters shown in (B). (D) Representative flow cytometry plots display the expression of CD44 and KLRG1 in M38_316–323_-specific CD8^+^ T cells. (E) Expression (gMFI) of PKM and CPT1a. Dots indicate individual mice (*n* = 7). Data are presented as mean ± SEM. Statistical analysis was performed by two-tailed unpaired t test. ∗∗∗∗*p* < 0.0001.

Another advantage of intracellular metabolic protein staining is that, aside from certain cell surface proteins such as CD98, most metabolic targets are cross-reactive between human and mouse species.^23^ To demonstrate the applicability of this approach in mice, we combined our established metabolic panel with murine phenotypic markers and MHC class I tetramers to identify MCMV-specific CD8^+^ T cells. Wild-type mice were infected with either a low (2 × 10^2^ plaque-forming units [PFU]) or high (2 × 10^4^ PFU) dose of MCMV. Unsupervised UMAP analysis of M38_316–323_-specific CD8^+^ T cells revealed distinct metabolic profiles between the two groups (Figures 5A–5E). In the low-dose group, MCMV-specific CD8^+^ T cells exhibited elevated expression of proteins associated with mitochondrial FAO (CPT1a), mitochondrial respiration (cytochrome c), and the pentose phosphate pathway (G6PD). In contrast, cells from the high-dose group showed a more activated effector-memory phenotype, marked by increased KLRG1 expression, and upregulation of the glycolysis-related proteins PKM and GLUT1 (Figures 5A–5E). These findings illustrate that the infectious dose shapes the metabolic profile of antigen-specific CD8^+^ T cells, reflecting differences in their activation and functional state.

### Fluorescent probes provide insight into functional metabolic properties of antigen-specific CD8^+^ T cells

To gain additional insight into metabolism at single-cell resolution, various affordable fluorescent probes correlating to different metabolic cellular activities and characteristics are available. Here, we used spectral flow cytometry to combine the uptake of 2-NBDG (indicating glucose uptake), BODIPY FL-C16 (indicating the uptake of long-chain fatty acids), MitoTracker Deep Red (MTDR; indicates mitochondrial mass), and TMRM (indicating mitochondrial membrane potential). The combined usage of these probes can be challenging on conventional flow cytometers due to the spectral overlap between the dyes. The combination with MHC class I tetramers further complicates combinatorial staining as tetramers are commonly available in conjugation to phycoerythrin (PE) or allophycocyanin (APC). The emission peak of PE (∼576 nm) and TMRM (∼574 nm) is nearly identical, which makes it impossible to combine staining on conventional flow cytometers, and even troublesome for spectral flow cytometers. Therefore, we recommend MHC class I tetramers conjugated with APC. Although the emission peak of APC (∼660 nm) is close to MTDR (∼665 nm), both signals can be distinguished by spectral flow cytometry. In contrast to the staining of the intracellular metabolic proteins such as PKM and ATP5a, staining with these probes do not require fixation. In fact, fixation can even perturb the staining. Representative FMO controls of the staining with BODIPY FL-C16 and 2-NBDG are shown in Figure S6A.

Another recommendation is to first assess the specificity of the different metabolic probes with controls (Figure S6B). Preferably, this should be performed for each cell population of interest and for each experimental set-up, as e.g., the incubation time of metabolic probes, different culture conditions, and cell size and number can all influence the results. For example, staining with probes is routinely performed in medium without fetal calf serum (FCS). The addition of 10% FCS, which contains fatty acids, results in a marked reduction in the uptake of BODIPY FL-C16. Moreover, the uptake of MTDR can be affected by the mitochondrial membrane potential, which makes this probe, in certain settings, less suitable to measure mitochondrial mass.^24^ In our settings, treatment with the mitochondrial OXPHOS uncoupler FCCP does not reduce the uptake of MTDR, indicating that MTDR staining is not dependent on the mitochondrial membrane potential. In contrast, we consistently found that the uptake of TMRM declines after FCCP treatment.^25^

To evaluate whether metabolic probe uptake is affected by cell number per sample, we measured TMRM and MTDR uptake at varying cell numbers. Across all tested dye concentrations, the staining intensity consistently varied with cell number (Figures S6C and S6D). Therefore, similar to metabolic protein staining, the uptake of metabolic probes is influenced by the number of cells in the sample.

To correct for batch effects, we tested the effect of the algorithm CytoNorm^26^ on the uptake of metabolic probes of a reference sample, which was used in three independent experiments (Figure S7A). Except for 2-NBDG staining, differences were observed between the three batches prior to normalization. However, normalization allowed us to achieve comparable uptake patterns across batches. A detailed description is provided in the STAR Methods section.

**Figure 6:**
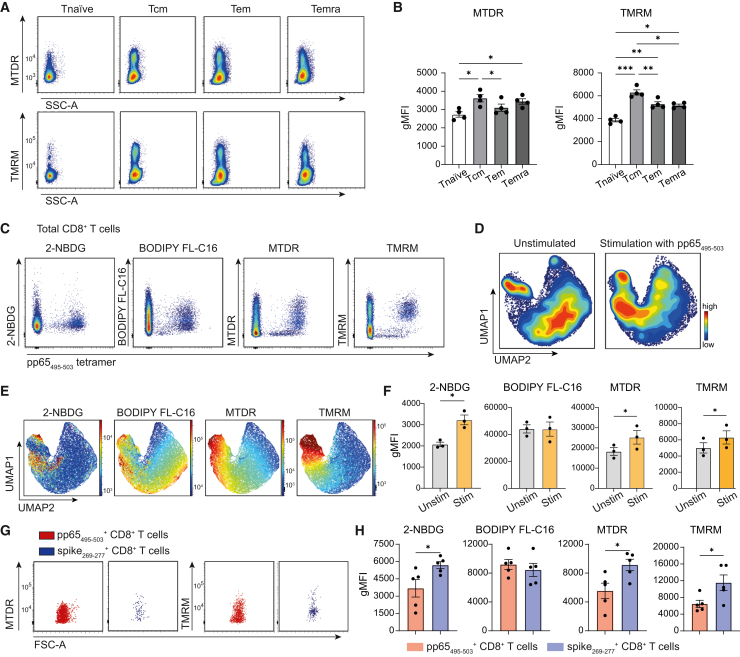
Fluorescent metabolic probes reveal functional metabolic properties of antigen-specific CD8^+^ T cells (A) Representative flow cytometry plots show the uptake of fluorescent metabolic probes in circulating CD8^+^ T cell subsets from a human donor. (B) Quantification of metabolic probe uptake (gMFI) in circulating CD8^+^ T cell populations from healthy donors (*n* = 4). (C) Representative flow plots showing the uptake of fluorescent metabolic probes by pp65_495–503_-specific CD8^+^ T cells. Gated on total CD8^+^ T cells. (D–F) Healthy HCMV-positive donor PBMCs were stimulated for 20 h with pp65_495–503_ peptide. (D) UMAP analysis on equally down-sampled pp65_495–503_-specific and CD137^+^ CD8^+^ T cells in unstimulated (left) and stimulated (right) conditions (*n* = 3). (E) Expression of fluorescent metabolic probes mapped onto the UMAP. (F) Quantification of metabolic probe uptake (gMFI) by antigen-stimulated vs. unstimulated pp65-specific CD8^+^ T cells. (G and H) PBMCs from donors with mild acute SARS-CoV-2 infection and positive CMV serology were analyzed. (G) Representative flow cytometry plots showing the uptake of fluorescent metabolic probes vs. FSC-A in pp65_495–503_-specific (red) and spike_269–277_-specific (blue) CD8^+^ T cells. (H) Quantification of metabolic probe uptake (gMFI) in pp65_495–503_-and spike_269–277_-specific CD8^+^ T cells (*n* = 5). Data are presented as mean ± SEM. Statistical analysis was performed using repeated-measures ANOVA with Tukey’s multiple comparisons test or two-tailed paired t tests. ∗*p* < 0.05; ∗∗*p* < 0.01, ∗∗∗*p* < 0.001. See also Figures S6 and S7 and Table S1.

The analysis of mitochondrial properties in human CD8^+^ T cell subsets revealed that T_cm_ cells displayed the highest mitochondrial mass (indicated by MTDR) and membrane potential (TMRM), emphasizing their higher mitochondrial activity (Figures 6A and 6B).^27^ T_em_ and T_emra_ cells also showed increased mitochondrial mass and membrane potential relative to T_naive_ cells, reflecting their ability to rapidly increase their bioenergetic machinery upon antigen re-challenge.^17^

Next, we combined the assay with MHC class I tetramers to demonstrate the uptake of metabolic probes by HCMV-specific CD8^+^ T cells (Figure 6C; Table S1). We then assessed the impact of a 20-h *in vitro* stimulation with the pp65_495–503_ peptide on metabolic probe uptake (Figures 6D and 6E; Table S1). Antigen-activated CD8^+^ T cells were identified by CD137 expression. We recorded a strong increase of 2-NBDG, TMRM, and MTDR uptake, but not of BODIPY FL-C16, by antigen-stimulated CMV-specific CD8^+^ T cells (Figures 6D–6F). A comparable pattern was observed when comparing HCMV- and SARS-CoV-2-specific CD8^+^ T cells in HCMV-seropositive individuals with acute SARS-CoV-2 infection (Figures 6G, 6H, and S7B). The latter results are also in line with the metabolic protein expression in these individuals, where we observed an intensified expression of metabolic proteins in SARS-CoV-2-specific CD8^+^ T cells (Figures 4 and S4). Thus, the combination of metabolic protein abundances and probe-based metabolic assays synergizes to provide in-depth insight into the metabolic features of antigen-specific T cells.

### Assessing the metabolic capacity of resting and stimulated antigen-specific CD8^+^ T cells by SCENITH

Extracellular flux analysis is an established method in immunometabolism to provide functional information about glycolysis and mitochondrial respiration. However, its application requires purified populations and high cell numbers.^12^^,^^28^ The recently published flow cytometry-based method SCENITH offers an alternative for functional information, which can also be applied to study rare cell populations.^15^ The method evaluates puromycin incorporation as an indirect readout of protein synthesis, which highly correlates with ATP production. By adding inhibitors of glycolysis (2-deoxy-D-glucose [2-DG]) or mitochondrial ATP synthase (oligomycin) or combined, it is possible to assess the impact of the blockade of each pathway on ATP production. The advantages of SCENITH include its reliance on a single fluorescent channel (to measure puromycin incorporation), which enables simultaneous staining with antibodies against phenotypic or metabolic markers, and the generation of data at single-cell resolution. Here, we combined SCENITH with MHC class I tetramer or CD137 staining. This provides the opportunity to study antigen-specific CD8^+^ T cell populations *ex vivo*, which is not feasible with extracellular flux analysis due to limited cell numbers.

**Figure 7:**
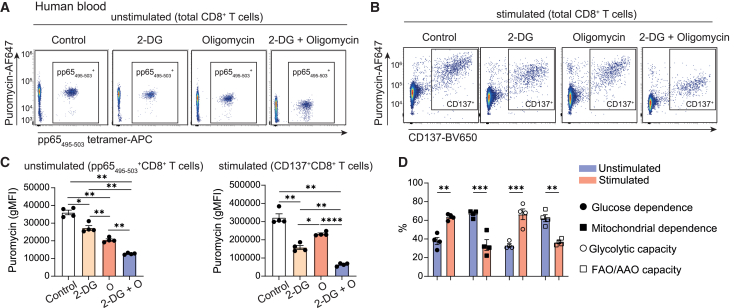
SCENITH-based assessment of metabolic capacity in resting and stimulated antigen-specific CD8^+^ T cells PBMCs from healthy HCMV-seropositive donors were left unstimulated or stimulated for 3 days with pp65_495–503_ peptide prior to performing the SCENITH assay (A) Representative flow cytometry plots showing puromycin incorporation in unstimulated pp65_495–503_-specific CD8^+^ T cells after treatment with vehicle (control), 2-DG, oligomycin, or both inhibitors. (B) Representative flow cytometry plots showing puromycin incorporation vs. CD137 expression in pp65_495–503_ peptide-stimulated CD8^+^ T cells under the same conditions as described in (A). (C) Quantification of puromycin incorporation (gMFI) in unstimulated pp65_495–503_-specific CD8^+^ T cells (left) and pp65_495–503_ peptide-stimulated CD137^+^CD8^+^ T cells (right; *n* = 4 donors). (D) SCENITH-derived metabolic parameters, i.e., glucose dependence, mitochondrial dependence, glycolytic capacity, and FAO/AAO capacity, were calculated for unstimulated pp65_495–503_-specific and pp65_495–503_ peptide-stimulated CD137^+^CD8^+^ T cells (*n* = 4 donors). Data are presented as mean ± SEM. Statistical analysis was performed by using repeated-measures ANOVA with Tukey’s multiple comparisons test or two-tailed paired Student’s t test. ∗*p* < 0.05; ∗∗*p* < 0.01; ∗∗∗*p* < 0.001; ∗∗∗∗*p* < 0.0001. See also Table S1.

We employed SCENITH to analyze the energetic metabolic properties of HCMV-specific CD8^+^ T cells in resting and activated states (Figures 7A–7D; Table S1). We selected a 3-day stimulation period to capture the activated state, as CD8^+^ T cells exhibit a robust activation phenotype at this time point. In the resting phase, the pp65_495–503_-specific CD8^+^ T cells showed high mitochondrial dependence and elevated FAO and amino acid oxidation (AAO) capacity (Figures 7A, 7C, and 7D). After stimulation with pp65 peptide for 3 days, activated HCMV-specific CD8^+^ T cells, as detected by the expression of CD137, displayed increased glucose dependence and glycolytic capacity, reflecting a changed metabolic requirement after activation in line with a higher energy demand (Figures 7B–7D). Of note, the CD137^+^ CD8^+^ T cells still displayed increased puromycin incorporation compared to the CD137^−^ CD8^+^ population in the 2-DG + oligomycin condition, which could be due to increased mitochondria and cell size after stimulation. However, compared to the control, it still leads to a drastic decline in puromycin incorporation. Taken together, the flow cytometry-based assays presented here highlight the possibility to specifically profile the energetic metabolism of antigen-specific T cell populations, as identified by MHC class I tetramers or CD137 expression.

## Discussion

The expanding interest in cellular metabolism, particularly in the context of chronic viral infections and cancer, has underscored the role of metabolic dysfunction in impaired CD8^+^ T cell responses. As a result, metabolic modulation is increasingly pursued to enhance T cell function.^6^^,^^7^^,^^9^ However, assessing the metabolism of antigen-specific CD8^+^ T cells remains technically challenging because of their availability in limited numbers. Here, we demonstrate how single-cell technologies, especially spectral flow cytometry, can integrate antigen specificity with diverse metabolic readouts. These approaches are also applicable to other rare immune cell subsets within heterogeneous, unpurified samples.

MHC class I multimer staining is the gold standard for *ex vivo* detection of antigen-specific CD8^+^ T cells without prior stimulation.^16^ We applied this method to detect virus-specific CD8^+^ T cells in human (HCMV and SARS-CoV-2) and murine (MCMV) samples, as well as tumor-specific CD8^+^ T cells in tumor tissue. While mass cytometry has extended multimer-based profiling,^29^ its low throughput limits broad application.^30^ Spectral flow cytometry offers a compelling alternative, combining high dimensionality with high throughput, enabling efficient metabolic profiling across large cohorts and experimental conditions.^30^^,^^31^ Using the Cytek Aurora, we demonstrated that metabolic protein markers and fluorescent probes can be reliably integrated with multimer staining. Comparable results were obtained using another spectral analyzer (i.e., SONY ID7000). Moreover, conventional flow cytometry can also be used. Data analysis was primarily performed with OMIQ.ai, though platforms such as FlowJo are equally compatible.

Spectral flow and mass cytometry enables the quantification of key metabolic proteins at single-cell resolution. As demonstrated by Hartmann et al., the abundance of metabolic proteins by mass cytometry reliably reflects metabolic function and correlates with functional assays.^13^ Recently, it was also shown how spectral flow cytometry enables *ex vivo* metabolic profiling of macrophages with comparable resolution but faster sample processing.^23^ Here, we applied spectral flow cytometry to profile human and murine antigen-specific CD8^+^ T cells using both multimers and CD137-based identification. Depending on the research question, panels can be tailored with >20 markers, including metabolic proteins and lineage markers. While the availability of pre-conjugated antibodies remains limited, custom conjugation kits facilitate flexible panel design.

The staining performance of metabolic antibodies and probes is sensitive to several experimental parameters, including cell number, sample preparation techniques, and composition of the staining mix. These factors can significantly influence the consistency and reproducibility of results, making the rigorous standardization of cell input and antibody preparation essential across all samples and experimental batches.^32^^,^^33^ To address potential batch effects, reference controls and normalization algorithms (e.g., CytoNorm) help mitigate batch effects. In scenarios involving low-yield samples, intra-sample reference populations, such as naive CD8^+^ T cells, can serve as an internal standard for normalization.^7^ These populations typically exhibit stable metabolic profiles, which enable researchers to adjust for technical variations, thereby improving the robustness of the data.

We also demonstrated that live-cell metabolic probes can be combined in multiplexed panels. The costs for probes are relatively low, and the readout can be performed by flow cytometry or imaging.^28^ Probes can indicate different metabolic properties of cells such as glucose uptake, long-chain fatty acid uptake, mitochondrial mass, and mitochondrial membrane potential, as well as mitochondrial-derived superoxide or lysosomal mass.^25^^,^^34^ Although 2-NBDG correlates with GLUT1, its uptake may not fully reflect glucose transporter activity, indicating the need for more specific probes.^35^ With the exception of MTDR, the other probes are not fixable and, therefore, difficult to combine with staining for intracellular metabolic proteins. Nonetheless, spectral flow cytometry allows the simultaneous assessment of at least four probes with multimers, streamlining analysis while conserving the sample.

Consistent with metabolic remodeling during T cell activation,^4^ we observed the upregulation of nutrient transporters (GLUT1 and CD98), glycolytic enzymes (PKM), and mitochondrial proteins (cytochrome c, SDHA, and ATP5a) in activated virus-specific CD8^+^ T cells. Moreover, *ex vivo* comparison of activated SARS-CoV-2- to HCMV-specific CD8^+^ T cells in the same host showed a comparable upregulation of different metabolic proteins in SARS-CoV-2-specific CD8^+^ T cells, which correlated with a higher percentage of cells expressing the T cell activation marker CD25. Interestingly, CPT1a was not significantly upregulated in activated SARS-CoV-2-specific CD8^+^ T cells, which aligns with the concept that activated CD8^+^ T cells rely less on FAO.^13^

Functional validation by the use of fluorescent metabolic probes underscored the protein-related data as increased glucose uptake and mitochondrial activity were observed after both the *in vitro* activation of HCMV-specific CD8^+^ T cells and *ex vivo* comparison between quiescent HCMV- and activated SARS-CoV-2-specific CD8^+^ T cells in SARS-CoV-2-infected individuals. In contrast, the uptake of fatty acids was largely similar in both the *in vitro* activation and *ex vivo* comparison. The assessment of energetic dependencies by SCENITH corroborated the previous results, as *in vitro* activated HCMV-specific CD8^+^ T cells displayed increased glucose dependence and glycolytic capacity and in turn decreased mitochondrial dependence and FAO/AAO capacity compared to their unstimulated counterparts.

In tissue-resident HCMV-specific CD8^+^ T cells, we noted elevated CPT1a and mitochondrial respiration markers, aligning with their FAO-dependent phenotype.^36^ Finally, we showed that the MCMV infection dose influenced CD8^+^ T cell metabolism, with high-dose infection promoting glycolysis and low-dose infection favoring mitochondrial FAO and respiration.

In summary, our approach leverages widely accessible flow cytometry platforms to enable multi-parametric metabolic profiling of antigen-specific CD8^+^ T cells, rare but critical populations for immunity, and useful as biomarkers of disease activity and therapeutic response. While we focused on virus-specific CD8^+^ T cells, the use of MHC class I tetramers and CD137-based identification extends this methodology to other pathogens, tumors, and autoimmune conditions, supporting broad applicability across diverse research areas.

### Limitations of the study

Flow cytometry-based profiling provides targeted insights into metabolic states but is inherently biased by probe and antibody selection. In contrast, omics approaches (e.g., transcriptomics and metabolomics) offer broader coverage but are currently constrained by cell number requirements, particularly for rare populations like antigen-specific T cells. Ongoing advances in omics technologies may eventually overcome these limitations, enabling more comprehensive profiling of scarce cell subsets at single-cell resolution.^12^ In addition, to ensure reliable and comparable results, where differences reflect true biological variation rather than technical artifacts, researchers must strictly adhere to standardizing protocols for cell counting, viability assessment, and antibody/probe dilution, while maintaining consistent incubation conditions throughout the experiment.

## Resource availability

### Lead contact

Further information and requests for resources and reagents should be directed to and will be fulfilled by the lead contact, Ramon Arens (r.arens@lumc.nl).

### Materials availability

This study did not generate new unique reagents.

### Data and code availability

All data reported in this manuscript will be shared by the lead contact upon request. This manuscript does not report original code. Any additional information to reanalyze the data reported in this paper is available from the lead contact upon request.

## Acknowledgments

N.M. received personal research funding from 10.13039/501100004426Dr. Werner Jackstädt-Stiftung (project no. S0134–10.124) and the 10.13039/501100001659German Research Foundation (project no. 526085745). The authors acknowledge the support of the 10.13039/501100005039LUMC Flow Cytometry Core Facility for their assistance with the operation of spectral flow cytometry.

## Author contributions

Conceptualization, N.M. and R.A.; methodology, N.M., J.F.d.G., G.A.H., B.E., and R.A.; investigation, N.M. and J.F.d.G; formal analysis, N.M. and J.F.d.G.; visualization, N.M., J.F.d.G., and R.A.; resources, K.B. and B.W.; writing – original draft, N.M. and R.A.; writing – review & editing, J.F.d.G., G.A.H., K.B., B.W., and B.E.; supervision, R.A. All authors read and approved the final manuscript.

## Declaration of interests

The authors declare no competing interests.

## STAR★Methods

### Key resources table

**Table:** 

REAGENT or RESOURCE	SOURCE	IDENTIFIER
**Antibodies**		

Antibodies and dyes for flow cytometry	This manuscript (Table S2)	N/A
αCD3 (1XE clone)	Hybridoma supernatant, kindly provided by Dr. Remmerswaal, Amsterdam UMC	N/A
αCD28 (clone CD28.2)	Biolegend	Cat#302902; RRID:AB_314304

**Chemicals, peptides, and recombinant proteins**		

Puromycin	Fisher Scientific	Cat#15480717
2-Deoxy-D-Glucose	Sigma Aldrich	Cat#D8375
Oligomycin	Abcam	Cat#ab141829
FCCP	Sigma Aldrich	Cat#C2920
NLVPMVATV peptide	Peptide and MHC-tetramer facility LUMC	N/A
Ficoll Paque PLUS	Cytiva	Cat#GE17-1440-02
Trypsin-EDTA (0.05%)	ThermoFisher Scientific	Cat#22409031
L-glutamine (200nM)	ThermoFisher Scientific	Cat#25030123
Penicillin-Streptomycin (10,000 U/ml)	ThermoFisher Scientific	Cat#15140122
Fetal calf serum	Bodinco BV	N/A
DNase I Type IV	Sigma Aldrich	Cat#D5025
Liberase™ TL Research Grade	Roche	Cat# 5401020001
Fc-block	BioLegend	Cat#422302
Dasatinib	Merck	Cat#SML2589

**Critical commercial assays**		

Foxp3/Transcription Factor Staining Buffer Set	eBioScience	Cat#00-5523-00
DyLight 405 conjugation kit – lightning kit	Abcam	Cat#ab201798
PE-Cy7 conjugation kit – lightning kit	Abcam	Cat#ab102903
PE-Cy5 conjugation kit – lightning kit	Abcam	Cat#ab102893
Alexa Fluor 647 conjugation kit – lightning kit	Abcam	Cat#ab269823
APC-Cy7 conjugation kit – lightning kit	Abcam	Cat#ab102859
PE conjugation kit – lightning kit	Abcam	Cat#ab102918
DyLight488 conjugation kit – lightning kit	Abcam	Cat#ab201799
Naive CD8^+^ T cell Isolation kit, human	Miltenyi	Cat#130-093-244

**Experimental models: Organisms/strains**		

Voluntary human blood donors	University Hospital Essen	N/A
Human spleen donors	Leiden University Medical Center	N/A
Buffy coats	Sanquin	N/A
C57BI/6J mice	Janvier labs	N/A

**Software and algorithms**		

OMIQ	OMIQ	https://www.omiq.ai/
GraphPad Prism software version 10.2.3	GraphPad Software	https://www.graphpad.com/
SpectroFlo version 3.3	Cytek Biosciences	https://cytekbio.com/
FlowJo v10.8.1	BD Life Sciences	www.flowjo.com/

**Other**		

IMDM	ThermoFisher Scientific	Cat#21980065
HLA-A∗02 tetramer HCMV pp65_495-503_ (NLVPMVATV)-APC	Peptide and MHC-tetramer facility LUMC	N/A
HLA-A∗01 tetramer HCMV pp65_363-373_ (YSEHPTFTSQY)-APC	Peptide and MHC-tetramer facility LUMC	N/A
HLA-A∗02 tetramer SARS-CoV-2 spike_269-277_ (YLQPRTFLL)-APC	Peptide and MHC-tetramer facility LUMC	N/A
H2-Kb tetramer MCMV M38_316-323_ (SSPPMFRV) APC	Peptide and MHC-tetramer facility LUMC	N/A
H-2Kb tetramer MuLV gp70 p15E (M8)_604-611_ (KSPWFTTL)-APC	Peptide and MHC-tetramer facility LUMC	N/A

### Experimental model and study participants details

#### Subject approval and participants

Adult individuals with acute SARS-CoV-2 infection and positive anti-CMV IgG serology were included at the outpatient clinic of the Department of Nephrology, University Hospital Essen, Germany. Other underlying diseases were not known. Acute SARS-CoV-2 infection was defined as positive PCR test within 14 days after symptom onset. Blood samples were shipped per overnight-express at room temperature to the Department of Immunology, Leiden University Medical Center. Immediately after arrival (within 24 h after blood donation), the samples were processed. Written informed consent was provided by all individuals and the study was approved by the local Ethics Committee of the University Hospital Essen, University of Duisburg Essen, Germany (16-7229-BO). Buffy coats from adult healthy, anti-CMV IgG positive individuals without acute SARS-CoV-2 infection were recruited through voluntary blood donation at Sanquin blood bank Amsterdam, the Netherlands. Splenic tissues from Dutch solid organ transplant donors were obtained during explant surgery as part of the diagnostic procedure for HLA typing in accordance with the Dutch law of organ donation.

Peripheral blood mononuclear cells (PBMCs) from buffy coats and lymphocytes from mechanically processed human spleen were isolated by Ficoll-Hypaque (GE Healthcare) density gradient centrifugation, and cryopreserved in liquid nitrogen until the day of analysis. Samples were acquired with ethical approval of the donors. Our study examined both men and women, with similar findings in both sexes.

#### *In vivo* infection model

C57BL/6J mice were intraperitoneally inoculated with murine cytomegalovirus (MCMV-Smith, obtained from the American Type Culture Collection, ATCC VR-194; Manassas, VA) (low dose: 2 × 10^2^ PFU; high dose: 2 × 10^4^ PFU). Mice were weighed 3 times per week, but no weight loss or other signs of discomfort were observed. On day 20 after MCMV infection, mice were sacrificed. Single-cell suspensions from the spleen were prepared by mincing the tissue through a 70 μm cell strainer. Contaminating erythrocytes were removed by ammonium chloride buffer. Animal experiments were approved by the Animal Experimental Committee of LUMC and performed according to the recommendations and guidelines set by LUMC and by the Dutch Experiments on Animals Act (permit number AVD1160020186804). We studied male and female animals, with comparable results across sexes.

### Method details

#### MHC class I tetramers

MHC class I tetramers were generated in-house and conjugated to APC. The following tetramer/peptide complexes were used to identify human virus-specific CD8^+^ T cells: HLA-A2/pp65_495-503_ (NLVPMVATV; HCMV), HLA-A1/pp65_363-373_ (YSEHPTFTSQY; HCMV) and HLA-A2/spike_269-277_ (YLQPRTFLL; SARS-CoV-2). To identify mouse virus-specific CD8^+^ T cells, H2-K^b^/M38_316-323_ (SSPPMFRV; MCMV) tetramer/peptide complexes were used.

#### Cell culture and stimulation with synthetic peptides

Frozen PBMCs or human spleen-derived lymphocytes were thawed in a 37°C water bath and washed in Iscove’s Modified Dulbecco’s Medium (IMDM), supplemented with 8% FCS (Bodinco), 100 U/ml penicillin (Gibco), 100 U/ml streptomycin (Gibco), 2 mM L-glutamine (Gibco), hereafter referred to as complete IMDM. Cells were centrifuged at 300 × g and subsequently rested in complete IMDM containing 30 μg/mL DNAse I type IV (Sigma) at 37°C and 5% CO_2_ for 2 h up to overnight to allow recovery prior to downstream procedures.

For *ex vivo* metabolic analysis, cells were washed twice with PBS before proceeding with staining protocols (described below). For stimulation of HCMV-specific CD8^+^ T cells, PBMCs were cultured in complete IMDM in presence of the HLA-A∗02-restricted HCMV pp65_495-503_ (NLVPMVATV) peptide epitope at a final concentration of 10 μg/mL.

As peptide stimulation leads to TCR downregulation and MHC class I tetramer internalization, but also induces expression of activation markers such as CD137 (4-1BB), the latter was used to identify antigen-reactive CD8^+^ T cells at early timepoints following stimulation.^22^ After 6 days of stimulation, expression of the TCR is re-established (via recirculation), allowing identification of pp65_495-503_-specific CD8^+^ T cells using MHC class I tetramers (i.e., HLA-A2/NLVPMVATV tetramers).

#### Isolation of tumor-infiltrating lymphocytes (TILs) from mice

Tumors were excised from mice following transcardial perfusion with 20 mL of 2 mM EDTA in PBS to remove circulating lymphocytes from the subcutaneous tumor. Tumor tissue was mechanically minced and subsequently subjected to enzymatic digestion using 0.1 mg/mL Liberase (Roche) for 30 min at 37°C. Next, the digested tissue was passed through a 70 μm cell strainer to obtain a single-cell suspension. Cells were counted using a hemocytometer and seeded at a density of 2×10^5^ cells per well for downstream staining and analysis.

#### Viability, cell surface and tetramer staining

Cultured cells were transferred to round-bottom 96-well plates at the desired cell concentration. For human splenocytes, Fc receptors were blocked by incubating cells with Fc-block (Biolegend) for 10 min at 4°C, followed by two washes with PBS. Viability staining was performed using Zombie NIR (BioLegend), Zombie Aqua (BioLegend) or Live Dead Fixable Blue (ThermoFisher) in PBS for 15 min at room temperature. MHC class I tetramer staining was performed in staining buffer (PBS supplemented with 1% FCS) for 30 min at 4°C. Subsequent cell surface staining, including for the amino acid transporter CD98, was performed using fluorescently-labeled antibodies for 30 min at 4°C. Tetramer staining was conducted prior to surface antibody staining to reduce TCR internalization by CD3 antibodies and to prevent interference with tetramer binding by CD8 antibodies.

#### Intracellular staining for metabolic proteins

To ensure reliable quantification of metabolic proteins, equal cell numbers across samples within each experiment were maintained (Figure S2E). Cell counts were performed on the day of flow cytometric analysis, including post-incubation or stimulation, as plating conditions may affect cell recovery. To ensure sufficient recovery of virus-specific CD8^+^ T cells for downstream analysis, 1×10^6^ cells were typically plated per well in 96-well round-bottom plates. Depending on the frequency of antigen-specific T cells, additional cells could be plated per well, or multiple wells pooled, to obtain adequate cell numbers for flow cytometric analysis.

Antibodies used for intracellular staining of metabolic proteins were conjugated in-house using Lightning-Link antibody labeling kits (Abcam), according to the manufacturer’s instructions. After completion of viability, tetramer, and surface marker staining, cells were fixed and permeabilized using the Fixation/Permeabilization reagent of the Foxp3/Transcription Factor Staining Buffer Set (eBioscience, Invitrogen) for 45 min at 4°C. Following two washes in Permeabilization Buffer, antibodies against intracellular metabolic proteins were added and cells were stained for 1 h at 4°C. Cells were washed twice more in Permeabilization Buffer prior to acquisition.

For experiments involving cell proliferation, naive human CD8^+^ T cells were isolated by magnetic-activated cell sorting (MACS) from PBMCs and subsequently labeled with CellTrace Violet (CTV; ThermoFisher Scientific). Cells were then stimulated with anti-CD3 and anti-CD28 antibodies for 6 days. On day 6, metabolic protein staining was performed as described above.

#### Staining with fluorescent metabolic probes

As with intracellular metabolic protein staining, reliable quantification of fluorescent metabolic probes requires equal cell numbers across all samples within each experiment (Figures S6C and S6D). Cell counts should be performed on the day of analysis, including after any incubation or stimulation steps, to ensure consistency and comparability.

Metabolic probe staining was performed following viability staining and prior to tetramer and cell surface staining to minimize potential T cell activation via TCR engagement. Cells were incubated in pre-warmed, FCS-free IMDM containing the respective metabolic dyes incubated for 30 min at 37°C and 5% CO_2_. Mitochondrial membrane potential was assessed by tetramethylrhodamine methyl ester (TMRM, ThermoFisher Scientific) at a final concentration of 4 nM. Mitochondrial mass was measured using MitoTracker Deep Red (ThermoFisher Scientific), also at a concentration of 4 nM.

To ensure that MitoTracker Deep Red staining is not affected by the mitochondrial membrane potential, cells were treated with the mitochondrial uncoupler FCCP.^25^ As expected, FCCP decreased the TMRM signal substantially, while the MitoTracker Deep Red signal minimally increased, likely due to mitochondrial swelling.

To assess glucose uptake, cells were incubated with the fluorescent glucose analog 2-(N-(7-nitrobenz-2-oxa-1,3-diazol-4-yl)amino)-2-deoxyglucose (2-NBDG, Life Technologies) at a concentration of 5 μM. Specificity of uptake was measured by co-incubation with the competitive inhibitor 2-deoxyglucose (2-DG), which results in a modest 2-NBDG signal reduction.

Long-chain fatty acid uptake was measured by staining with 4,4-Difluoro-5,7-Dimethyl-4-Bora-3a,4a-Diaza-*s*-Indacene-3-Hexadecanoic Acid (BODIPY FL C_16_, ThermoFisher Scientific) at a concentration of 25 nM. Specificity was confirmed by co-incubation with 10% FCS, which reduced BODIPY FL C_16_ uptake due to competition by serum-derived lipids.

Normalization procedures for comparing metabolic probe staining across experimental batches are described in the “Acquisition and data analysis” section below. Note that normalization procedures can also be applied for comparing metabolic protein staining.

#### SCENITH

Human PBMCs were either directly subjected to SCENITH or first stimulated with pp65_495-503_ peptide (NLVPMVATV) for 3 days followed by SCENITH. SCENITH was performed following the original published protocol.^15^ Briefly, cells were incubated for 20 min at 37°C, 5% CO_2_ with either control medium (CTL), 100 mM 2-DG, 1 μM oligomycin (O), or a sequential combination of both inhibitors (2-DG + O). Subsequently, puromycin (10 μg/mL) was added for an additional 20 minutes. After treatment, cells were washed, and stained for viability, followed by tetramer and cell surface staining as described above. Next, cells were fixed and permeabilized using the eBioscience Foxp3/Transcription Factor Staining Buffer Set (Invitrogen), and intracellular puromycin incorporation was detected using a monoclonal anti-puromycin antibody (Puro) for 45 minutes at 4°C.

Metabolic dependencies and capacities were calculated based on geometric MFI of puromycin (PuroMFI) using the following equations:

$$\text{Glucose}\;\text{dependence}:100\times \left({\text{CTL}}^{\text{PuroMFI}}{-2-\text{DG}}^{\text{PuroMFI}}\right)/\left({\text{CTL}}^{\text{PuroMFI}}-{\left[2-\text{DG}+O\right]}^{\text{PuroMFI}}\right)$$

$$\text{Mitochondrial}\;\text{dependence}:100\times \left({\text{CTL}}^{\text{PuroMFI}}{-O}^{\text{PuroMFI}}\right)/\left({\text{CTL}}^{\text{PuroMFI}}-{\left[2-\text{DG}+O\right]}^{\text{PuroMFI}}\right)$$

$$\text{Glycolytic}\;\text{capacity}:100-100\times \left({\text{CTL}}^{\text{PuroMFI}}\;{-O}^{\text{PuroMFI}}\right)/\left({\text{CTL}}^{\text{PuroMFI}}-{\left[2-\text{DG}+O\right]}^{\text{PuroMFI}}\right)$$

$$\text{FAO}\;\text{and}\;\text{AAO}\;\text{capacity}:100-100\times \left({\text{CTL}}^{\text{PuroMFI}}{-2-\text{DG}}^{\text{PuroMFI}}\right)/\left({\text{CTL}}^{\text{PuroMFI}}-{\left[2-\text{DG}+O\right]}^{\text{PuroMFI}}\right)$$

#### Acquisition and data analysis

Except for Figure S6D (data acquired using a BD LSR Fortessa), all flow cytometry data were acquired using a Cytek Aurora 5-laser spectral analyzer equipped with SpectroFlo acquisition software (v3, Cytek). Reference controls for spectral unmixing were stained in parallel with the experimental samples, using either cells or compensation beads depending on marker brightness. Autofluorescence extraction was applied to enhance spectral unmixing accuracy.

Data were analyzed using OMIQ (OMIQ.ai) or FlowJo software. In OMIQ, parameters were scaled using conversion factors between 6000 and 10000, followed by gating on virus-specific CD8^+^ T cells. A representative gating strategy is shown in Figure S1A. High-dimensional analysis was performed by subsampling equal numbers of antigen-specific CD8^+^ T cells, followed by UMAP and FlowSOM consensus meta-clustering or clustered heatmap visualization using default settings.

To normalize metabolic probe staining across different experimental batches (see Figure S7A), a reference PBMC sample from the same donor (isolated and cryopreserved on the same day) was included in each experiment and stained alongside the test samples. Given the sensitivity of metabolic probe readouts to exact cell numbers and pipetting accuracy, even standardized protocols can result in variability between independent experiments. To address this, CytoNorm^26^ was used in OMIQ to correct for batch effects. After gating on virus-specific CD8^+^ T cells, FlowSOM clustering was applied to all relevant markers. All samples from each batch, including reference samples, were selected and assigned accordingly for normalization. CytoNorm was then applied to normalize marker expression across all samples based on the clustering results and reference sample profiles.

### Quantification and statistical analysis

Unless otherwise indicated, data are presented as mean ± SEM, with each symbol representing an individual sample. Statistical analyses were performed using GraphPad Prism (version 10.2.3). Comparisons between two independent groups were performed using two-tailed unpaired Student’s t-tests, and for comparisons within the same sample or subject two-tailed paired Student’s t-tests were used. For comparisons across more than two groups, repeated measures ANOVA with Tukey’s multiple comparisons test was used. *p*-values <0.05 were considered statistically significant and are indicated as follows: ∗*p* < 0.05, ∗∗*p* < 0.01, ∗∗∗*p* < 0.001, ∗∗∗∗*p* < 0.0001.

## Supplemental information

Document S1. Figures S1–S7 and Tables S1 and S2

Document S2. Article plus supplemental information
