# Supplementary material for: Metabolic profiling of antigen-specific CD8+ T cells by spectral flow cytometry
Source: Cell Rep Methods. 2025 Sep 26;5(10):101185. doi: 10.1016/j.crmeth.2025.101185 (PMC12570331; doi:10.1016/j.crmeth.2025.101185)
Supplement: Document S1. Figures S1–S7 and Tables S1 and S2 [file mmc1.pdf]

Cell Reports Methods, Volume *5*

Supplemental information

Metabolic profiling of antigen-specific CD8$^+$ T cells by spectral flow cytometry

Nils Mülling, J. Fréderique de Graaf, Graham A. Heieis, Kristina Boss, Benjamin Wilde, Bart Everts, and Ramon Arens

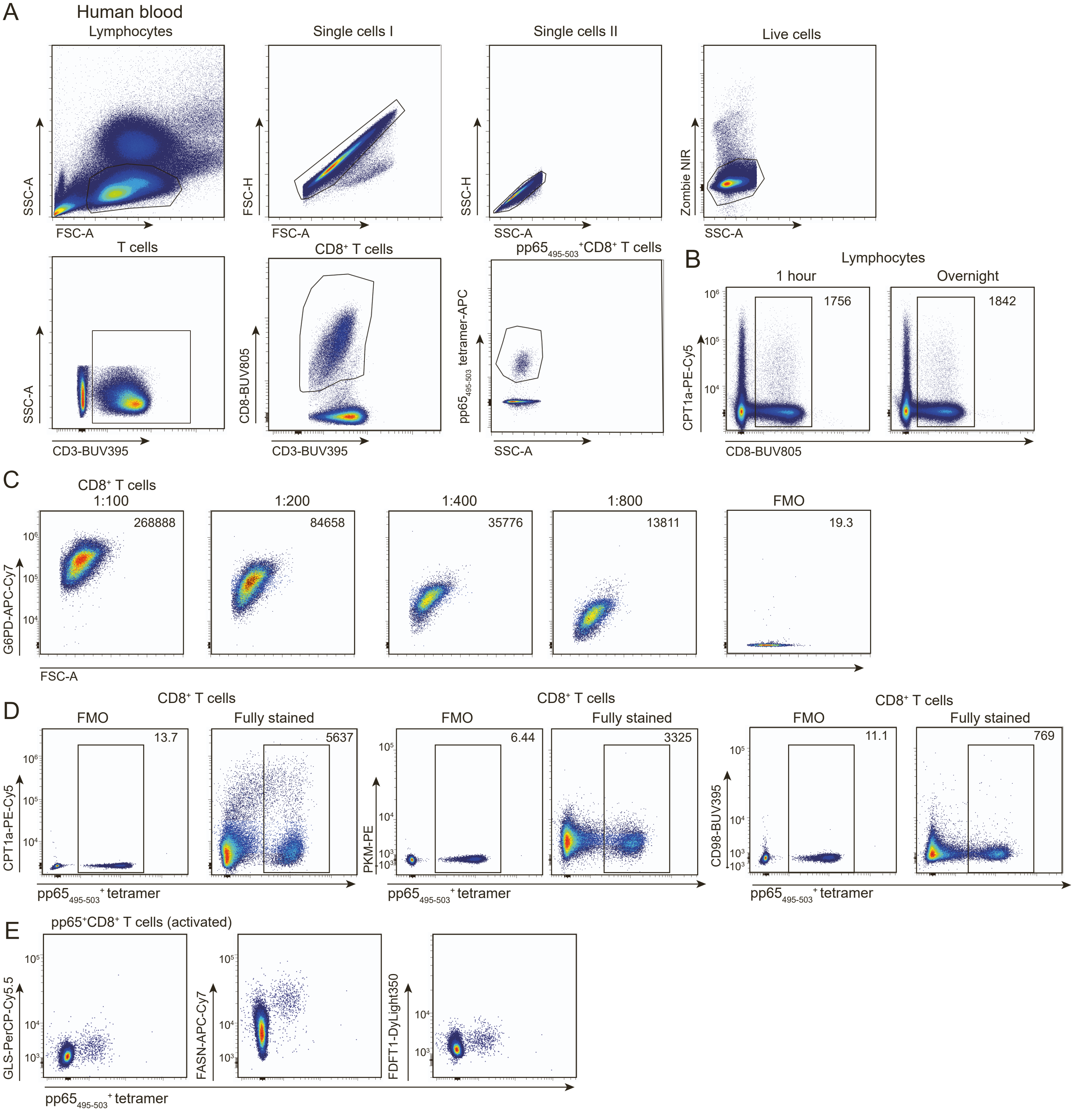

Figure S1. Gating strategies and quality controls for metabolic protein profiling in human CD8⁺ T cells, related to Figure 2.
(A) Representative flow cytometry gating strategy for the detection of MHC class I tetramer-positive CD8⁺ T cells (shown for $pp65_{495\text{-}503}$) in PBMCs from a human donor.
(B) Comparison of staining intensity after different resting times. PBMCs were thawed and rested for 1 hour or overnight prior to staining. Shown is staining for CPT1a in live lymphocytes.
(C) Example titration of antibodies targeting metabolic proteins. Gated on circulating live total CD8⁺ T cells from a human donor. Numbers indicate geometric mean fluorescence intensity (gMFI).
(D) Representative flow cytometry plots of fully stained samples and corresponding fluorescence-minus-one (FMO) controls. Gated on circulating live total CD8⁺ T cells. Boxes indicate gates on $pp65_{495\text{-}503}^{+}$CD8⁺ T cells. Numbers indicate gMFI.
(E) PBMCs were stimulated with $pp65_{495\text{-}503}$ peptide for 6 days. Representative flow cytometry plots show staining for glutaminase (GLS), fatty acid synthase (FASN) and farnesyl-diphosphate farnesyltransferase 1 (FDFT1). Gated on live CD8⁺ T cells.

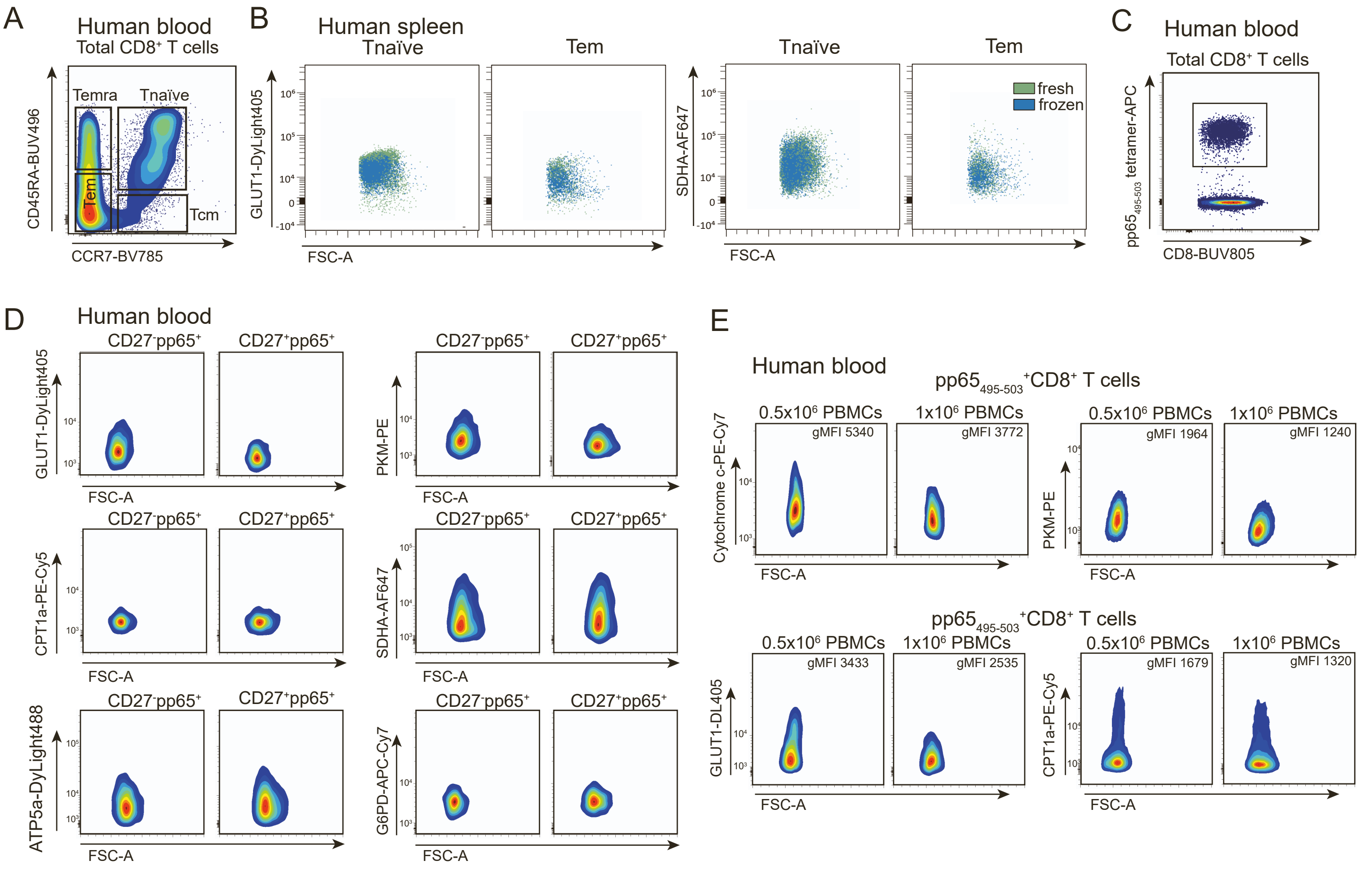

Figure S2. Validation of metabolic protein expression measurements, related to Figure 2.
(A) Representative flow cytometry plot showing the gating strategy of circulating $CD8^+$ T cell subsets in a human donor.
(B) Comparison of metabolic marker expression in fresh *versus* frozen samples. Lymphocytes were isolated from human spleen and either directly stained (fresh) or cryopreserved and rested overnight after thawing (frozen). Representative plots display GLUT1 and SDHA expression in Tnaive and Tem populations.
(C) Representative flow cytometry plot illustrating the gating of $pp65_{495\text{-}503}$-specific $CD8^+$ T cells in a human donor, identified using the MHC class I tetramer HLA-A*02-HCMV $pp65_{495\text{-}503}$ (NLVPMVATV). The population was pre-gated on $CD8^+$ T cells.
(D) Representative contour plots showing expression of metabolic proteins in $CD27^+$ and $CD27^-$ $pp65_{495\text{-}503}$-specific $CD8^+$ T cells from human blood.
(E) Representative flow cytometry plots demonstrating the effect of total input cell number on staining intensity (gMFI) of Cytochrome c, GLUT1, PKM and CPT1a in $pp65_{495\text{-}503}{}^+CD8^+$ T cells from human blood.

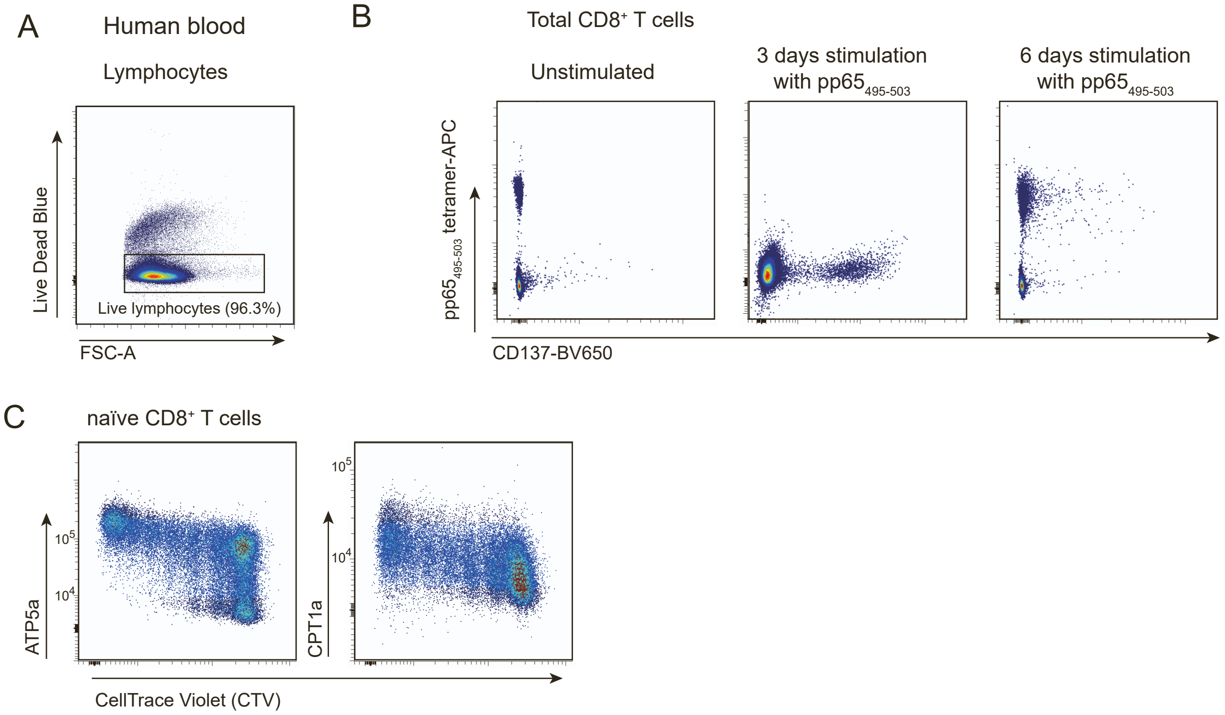

Supplemental Figure S3. Metabolic protein expression in stimulated CD8^{+} T cells, related to Figure 3.
(A) Representative flow cytometry plot showing viability of human lymphocytes after six days of culture.
(B) Representative flow cytometry plots showing the MHC class I tetramer (pp65$_{495\text{-}503}$) staining *versus* CD137 expression in total CD8^{+} T cells under different conditions: unstimulated, after 3 days stimulation with cognate antigen (pp65$_{495\text{-}503}$ peptide), and after 6 days of stimulation with cognate antigen.
(C) Naïve CD8^{+} T cells were isolated, labelled with CellTrace violet (CTV), and stimulated with for 6 days with anti-CD3 and anti-CD28 monoclonal antibodies. Representative flow cytometry plots show expression of ATP5a and CPT1a *versus* CTV.

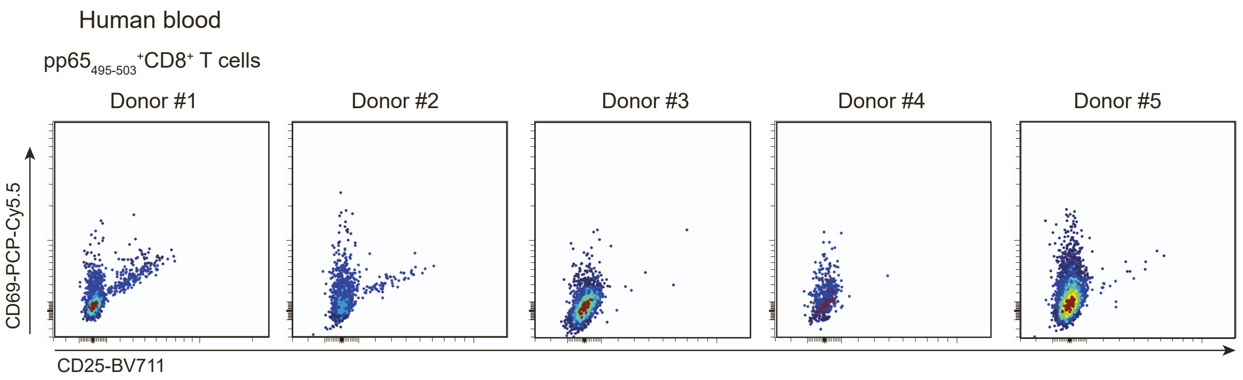

Figure S4. Metabolic profiling detects differences between antigen-specific CD8⁺ T cell populations directed against distinct viruses in the same host, related to Figure 4.
Blood from human volunteers with mild acute SARS-CoV-2-infection and positive HCMV serology was analyzed. Flow cytometry plots display expression of CD25 and CD69 in $pp65_{495\text{-}503}{}^{+}CD8^{+}$ T cells from all individual donors.

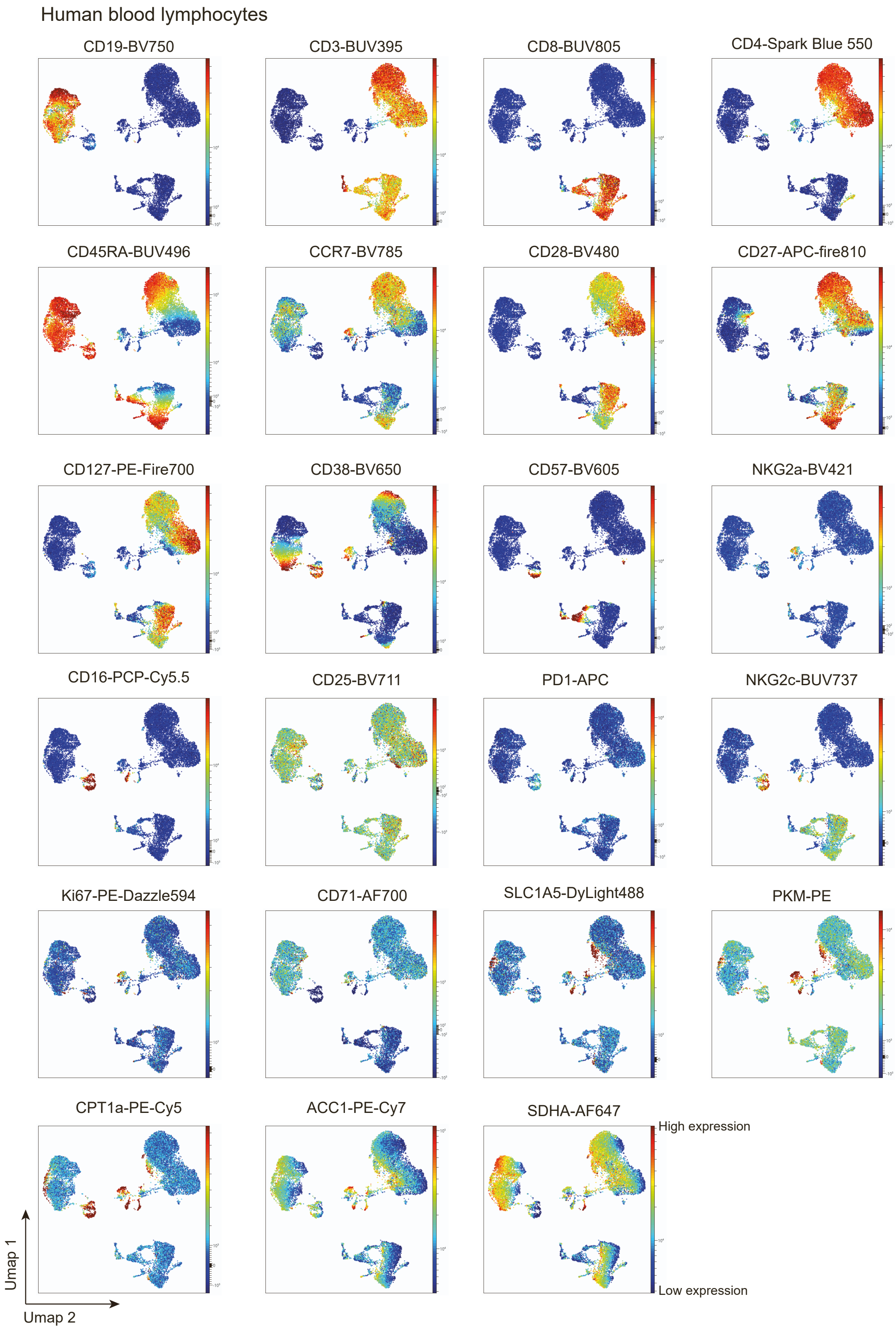

Figure S5. Example of a large spectral flow cytometry panel incorporating phenotypic and metabolic profiling of immune cells in PBMCs from a healthy donor, related to Figure 4.
UMAP plots show the expression of various phenotypic and metabolic proteins, gated on live lymphocytes. Blue indicates low expression; red indicates high expression.

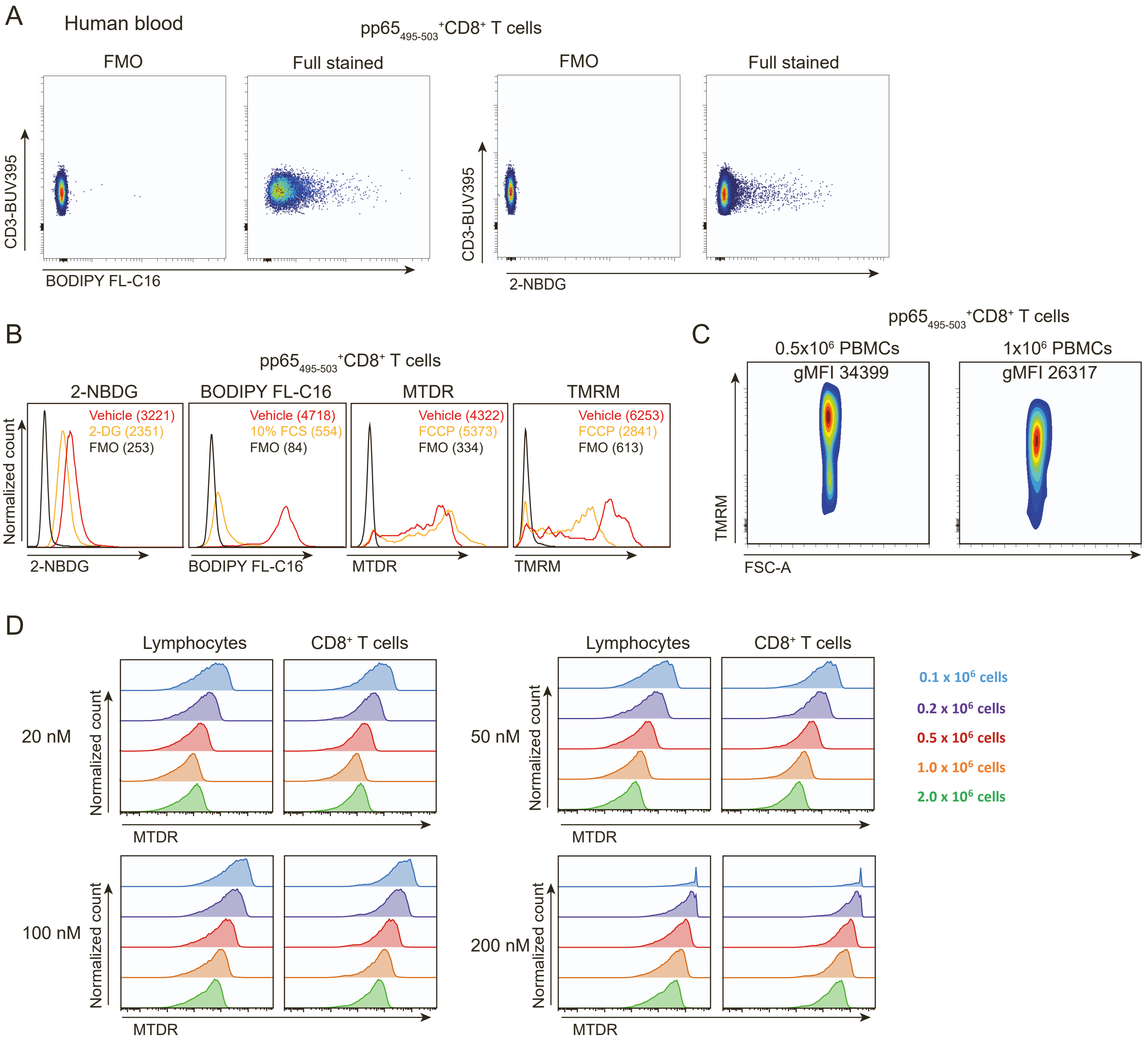

Figure S6. Recommendations for optimized metabolic profile measurements with fluorescent probes, related to Figure 6.
(A) Representative flow cytometry plots of fully stained samples and fluorescence-minus-one (FMO) controls. Gated on $pp65_{495\text{-}503}{}^{+}CD8^{+}$ T cells.
(B) Uptake of different fluorescent metabolic probes (2-NBDG, BODIPY FL-C16, MitoTracker Deep Red (MTDR), TMRM) by $pp65_{495\text{-}503}$-specific $CD8^{+}$ T cells. Cells were treated with either vehicle (red), a specific inhibitor or control (orange) (2-DG, 10% FCS, FCCP), or stained as FMO control (black).
(C) Representative flow cytometry plots showing the impact of total cell number on staining intensity (gMFI) of TMRM in $pp65_{495\text{-}503}{}^{+}CD8^{+}$ T cells.
(D) Histograms showing the effects of varying cell numbers (indicated by color) and different concentrations of MTDR on staining intensity. Gated on lymphocytes (left) and total $CD8^{+}$ T cells (right). Data acquired using a conventional flow cytometer (BD LSRFortessa), and analyzed using FlowJo.

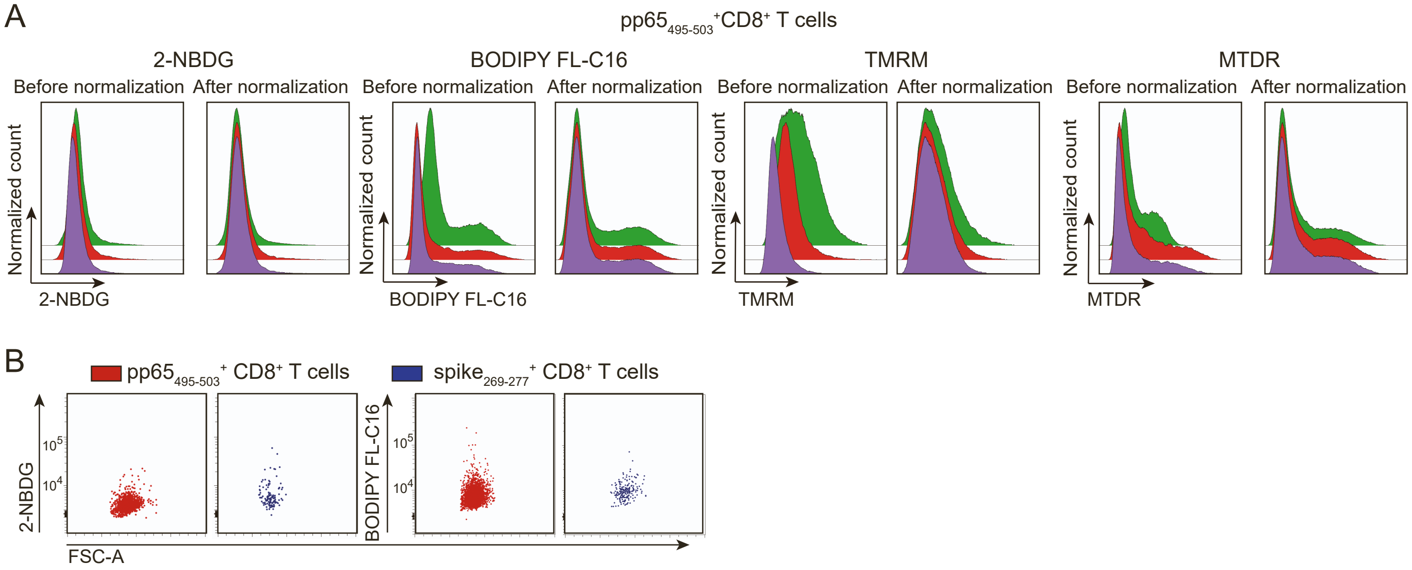

Figure S7. Recommendations for optimized metabolic profile measurements with fluorescent probes, related to Figure 6.

(A) Histograms show the uptake of different fluorescent metabolic probes by $pp65_{495\text{-}503}$-specific $CD8^+$ T cells from a healthy donor. Different colors represent independent experiments performed on different days using samples from the same donor (all samples were processed and cryopreserved on the same day). Before normalization: Staining data for each batch prior to normalization. After normalization: staining data following CytoNorm batch normalization.

(B) Representative flow cytometry plots display the uptake of different fluorescent metabolic probes plotted versus FSC-A for $pp65_{495\text{-}503}$ (red) and $spike_{269\text{-}277}$-specific (blue) $CD8^+$ T cells.

Table S1: Metabolic flow cytometry panels, related to Figures 2, 6, 7 and S5.

**Metabolic protein expression** (referring to Figure 2C, 2D)	
**Target**	**Fluorophore**
CD3	BUV395
CD8a	BUV805
CD27	APC-fire810
CD38	BV650
$pp65_{495\text{-}503}$ tetramer	APC
Live-Dead Fixable Blue	Blue-Fluorescent (~450 nm)
GLUT1	DyLight 405
PKM	PE
G6PD	APC-Cy7
CPT1a	PE-Cy5
ATP5a	DyLight 488
SDHA	Alexa Fluor 647
**Metabolic probes** (referring to Figure 6)	
**Target**	**Fluorophore**
CD3	BUV395
CD8a	BUV805
CD137	BV650
$pp65_{495\text{-}503}$ tetramer	APC
Live-Dead Fixable Blue	Blue-Fluorescent (~450 nm)
2-NBDG	
BODIPY FL-C16	
TMRM	
MitoTracker Deep Red	
**SCENITH** (referring to Figure 7)	
**Target**	**Fluorophore**
CD3	BUV395
CD8a	BUV805
CD137	BV650
$pp65_{495\text{-}503}$ tetramer	APC
Puromycin	Alexa Fluor 647

**Phenotyping and metabolic panel** (referring to Figure S5)	
**Target**	**Fluorophore**
CD19	BV750
CD3	BUV395
Live-Dead Fixable Blue	Blue-Fluorescent (~450 nm)
CD8a	BUV805
CD4	Spark Blue 550
CD45RA	BUV496
CCR7	BV785
CD28	BV480
CD27	APC-fire810
CD127	PE-Fire700
CD38	BV650
CD57	BV605
NKG2a	BV421
CD16	PerCP-Cy5.5
CD25	BV711
PD1	APC
NKG2c	BUV737
Ki67	PE-Dazzle594
CD71	AF700
SLC1A5	DyLight488
PKM	PE
CPT1a	PE-Cy5
ACC1	PE-Cy7
SDHA	AF647

Table S2: List of used antibodies, viability probes and fluorescent metabolic dyes for flow cytometry. Related to STAR Methods.

Target	Species	Fluorophore	Clone	Dilution	Supplier	Catalogue number
CD3	H	V450	UCHT1	1:50	BD Biosciences	560365
CD3	H	BUV395	UCHT1	1:100	BD Biosciences	563548
CD8a	H	BUV805	SK1	1:400	BD Biosciences	612889
CD8a	H	APC-fire810	SK1	1:1600	BD Biosciences	344764
CD19	H	BV750	HIB19	1:100	Biolegend	302245
CD4	H	Spark Blue 550	SK3	1:400	Biolegend	344655
CD28	H	BV480	CD28.2	1:50	BD Biosciences	566110
CD127	H	PE-Fire700	A019D5	1:100	Biolegend	351365
CD57	H	BV605	QA17A04	1:600	Biolegend	393303
NKG2a (CD159a)	H	BV421	131411	1:100	BD Biosciences	747924
CD16	H	PerCP-Cy5.5	3G8	1:75	Biolegend	302027
PD1 (CD279)	H	APC	EH12.2H7	1:200	Biolegend	329907
NKG2c (CD159d)	H	BUV737	134591	1:100	BD Biosciences	749685
Ki-67	H	PE-Dazzle594	Ki-67	1:200	Biolegend	350533
CD71	H	AF700	M-A712	1:200	BD Biosciences	563769
CD45RA	H	BUV496	HI100	1:1600	BD Biosciences	750258
CCR7	H	BV785	G043H7	1:50	BioLegend	353230
CD25	H	BV711	M-hla	1:200	BioLegend	356138
CD27	H	APC-fire810	QA17A18	1:200	BioLegend	393214
CD69	H	PerCP-Cy 5.5	FN50	1:50	Biolegend	310925
CD38	H	BV650	HIT2	1:50	Biolegend	569391
CD137	H	BV650	4B4-1	1:50	Biolegend	309828
CD8a	M	BUV395	53-6.7	1:600	BD Biosciences	565968
CD44	M	BUV805	IM7	1:800	BD Biosciences	741921
CD62L	M	BV711	MEL-14	1:2000	Biolegend	104445
KLRG1	M	BV785	2F1	1:100	Biolegend	138429
CD98	H	BUV395	UM7F8 (RUO)	1:400	BD Biosciences	744508
GLUT1	H/M	BSA/Azide free DyLight 405	EPR3915	1.4 ng/ul	Abcam	ab252403 ab201798
SLC1A5	H	Unconjugated DyLight 488	W19125A	8.3 ng/ul	Biolegend	608052 ab236553
ACC1	H/M	BSA/Azide free PE-Cy7	EPR23235-147	2.1 ng/ul	Abcam	ab272704 ab102903
CPT1a	H/M	BSA/Azide free PE-Cy5	EPR21843-71-2F	1.4 ng/ul	Abcam	ab235841 ab102893
Cytochrome C	H/M	BSA/Azide free PE-Cy7	7H8.2C12	1.4 ng/ul	Abcam	ab237966 ab102903
SDHA	H/M	BSA/Azide free Alexa Fluor 647	EPR9043(B)	0.42 ng/ul	Abcam	ab240098 ab269823

G6PD	H/M	BSA/Azide free APC-Cy 7	EPR20668	1.0 ng/ul	Abcam	ab231828 ab102859
PKM	H/M	BSA/Azide free PE	EPR10138(B)	2.1 ng/ul	Abcam	ab206129 ab102918
ATP5a	H/M	BSA/Azide free DyLight 488	EPR13030(B)	1.4 ng/ul	Abcam	ab231692 ab201799
FDFT1	H/M	DyLight 350	OTI2F10	8.3 ng/ul	Novus Biologicals	NBP2-70715R
Glutaminase	H/M	BSA/Azide free PE-Cy 7	EPR19525	1.4 ng/ul	Abcam	ab223129 ab102903
FASN	H/M	BSA/Azide free APC-Cy7	EPR7466	1.0 ng/ul	Abcam	Ab221934 Ab102859
Puromycin	H/M	Alexa Fluor 647	2A4	4.2 ng.ul	Biolegend	381507
**Cell viability probe**	**Species**		**Supplier**		**Catalogue number**	
Zombie NIR	H/M		BioLegend	1:1000	423106	
Live-Dead Fixable Blue	H/M		Thermo Fisher	1:500	L23105	
Zombie Aqua	H/M		BioLegend	1:1000	423102	
**Cell tracing dye**	**Species**		**Supplier**		**Catalogue number**	
CellTrace Violet	H/M		Thermo Fisher	1:1000	C34557	
**Fluorescent metabolic probe**	**Species**		**Supplier**	**Final concen-tration**	**Catalogue number**	
2-NBDG	H/M		ThermoFisher	50 µM	N13195	
BODIPY FL-C16	H/M		ThermoFisher	30 nM	D3821	
TMRM	H/M		ThermoFisher	5 nM	T668	
MitoTracker Deep Red	H/M		ThermoFisher	5 nM 20nM 200nM	M22426	
